# Not one-size-fits-all: µ-FTIR and pyrolysis GC-MS for complementary analysis of microplastics in eutrophic surface water

**DOI:** 10.1007/s00216-026-06446-w

**Published:** 2026-03-22

**Authors:** Timothy Omara, Barbora Benetková, Ivan Sumerskii, Patrick Ssebugere, Christine Kyarimpa, Solomon Omwoma Lugasi, Thomas Rosenau, Christine Betty Nagawa, Stefan Böhmdorfer

**Affiliations:** 1 Institute of Chemistry of Renewable Resources, Department of Natural Sciences and Sustainable Resources, BOKU University, Konrad-Lorenz-Straße 24, 3430 Tulln, Austria; 2 Department of Chemistry, College of Natural Sciences, Makerere University, P.O. Box 7062, Kampala, Uganda; 3 Core Facility Analysis of Lignocellulosics (ALICE), BOKU University, Konrad-Lorenz-Straße 24, 3430 Tulln, Austria; 4 Department of Chemistry, Faculty of Science, Kyambogo University, P.O. Box 1, Kampala, Uganda; 5 Department of Physical Sciences, Jaramogi Oginga Odinga University of Science and Technology, P.O. Box 210-40601, Bondo, Kenya; 6 Johan Gadolin Process Chemistry Centre, Åbo Akademi University, Porthansgatan 3, 20500 Åbo/Turku, Finland; 7 Department of Forestry, Biodiversity and Nature Conservation, College of Agricultural and Environmental Sciences, Makerere University, P.O. Box 7062, Kampala, Uganda

**Keywords:** Lake Victoria, Microplastic, Nylon, Polyethylene, Pyrolysis

## Abstract

This study reports large microplastics found in Lake Victoria through an analytical workflow that combines the complementary methods stereomicroscopy, micro-Fourier transform infrared (µ-FTIR) spectroscopy and pyrolysis gas chromatography-mass spectrometry (Pyr-GC-MS) for the quantification of 11 environmentally relevant microplastic polymers. Algae-rich surface water samples (*n* = 18) were trawled using a 0.3 mm manta net from Lake Victoria, the world’s largest tropical lake. Stereomicroscopy as a determinant analytical technique detected 191 particles, which were primarily blue fragments and fibres. Polyethylene and polypropylene were the dominant polymers identified by µ-FTIR. Pyr-GC-MS allowed the detection and quantification of microplastics (MPs) with LOD and LOQ of 0.01–14.7 µg and 0.03–49.1 µg. Polyethylene (0.058–0.34 µg/L), polypropylene (0.024 µg/L and 0.043 µg/L), nylon 6 (0.0051–0.064 µg/L), nylon 66 (0.0022–0.084 µg/L), poly(ethylene terephthalate) (0.0029–0.027 µg/L) and poly(methyl methacrylate) (0.0036 µg/L) were quantified. µ-FTIR was found to be suitable for the identification of the most abundant polymers in the 0.3–4.9 mm size range whereas Pyr-GC-MS afforded the quantification of seven polymers, most of which were not detected by µ-FTIR. This complementary workflow gave a wider perspective on MP loading, providing both polymer concentrations and physical characteristics (sizes, colours, forms and count) of the MPs.

**Graphical abstract:**

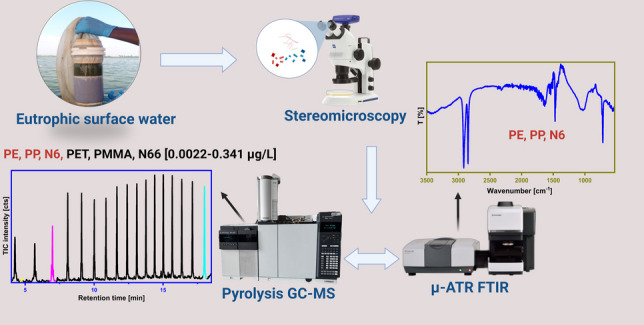

**Supplementary Information:**

The online version contains supplementary material available at 10.1007/s00216-026-06446-w.

## Introduction

Plastics are synthetic and semi-synthetic materials that have become emblematic of the single-use consumer culture. Since their discovery, plastics have replaced traditional materials, such as wood, ceramics and leather, and a world without plastics is to date inconceivable [1]. Beneath the facade of convenience associated with single-use plastics lies a considerably widespread and escalating concern—environmental plastic pollution. Plastic wastes, combined with the direct role of primary plastic production in releasing greenhouse gases, contribute without doubt to biodiversity loss, ecosystem degradation and the transit of harmful environmental contaminants [2]. One of the most concerning categories of plastic wastes are those with at least one of their dimensions being in the 1–1000 μm size range, the so-called microplastics, or ‘large microplastics’ for particles with dimensions of 1000–5000 μm [3].

Microplastic pollution is a global concern, and Africa is the continent with some of the most polluted water bodies [4]. This is partly due to laxity and the complexities associated with enforcing environmental regulations in lentic water resources [5]. Lake Victoria, the largest tropical lake, is a prime example of a shared freshwater resource in Africa which discharges its water into the Mediterranean Sea through the Nile River. The lake is recognised as a pollution hotspot with startling contamination by discharges from six countries within the East African Community, which has triggered eutrophication and accumulation of harmful algal blooms [6]. Microplastic contamination of water, fish and sediments from Lake Victoria is known [4, 7, 8], but limited understanding exists on the spatiotemporal scale of this pollution, especially with regard to the presence of cities, industries, fish landing beaches (FLBs) and other anthropogenic activities on its shores. Previous studies performed one-time sampling, providing particle counts, colours, sizes and polymer composition of MPs obtained by stereomicroscopy and micro-Fourier transform infrared (µ-FTIR) spectroscopy. These particle-based methods have inherent size limitations and are subject to substantial uncertainties [9].

To effectively track and mitigate mass-related MPs pollution in Lake Victoria, geospatial and temporal data are required. The best approach is to implement particle-based analytical techniques sequentially, alongside quantitative pyrolysis gas chromatography-mass spectrometry (Pyr-GC-MS), as has been demonstrated recently [10]. Thermoanalytical Pyr-GC-MS remains one of the most suitable techniques for qualitative and quantitative analysis of MPs. Its effectiveness is only challenged by the complex composition of real-world samples, as for example by the presence of multiple, weathered and structurally degraded polymers, variability in the pyrolytic breakdown and yield of pyrolysates and spectral interferences from inorganic and organic matter [9]. Further, secondary gas-phase reactions tend to occur whenever multiple polymers are present in a sample, thereby suppressing or enhancing signal intensity of pyrolysates and complicating polymer quantification [11, 12].

This study investigated the spatiotemporal dynamics of MPs in surface water from Lake Victoria. The majority of the trawled samples contained algae with mucilaginous exudates, suspended particulate matter and a high concentration of organic matter. For this reason, a careful combination of MP-extraction protocol and analytical techniques was chosen. Density separation of MPs was achieved with zinc chloride solution which helped to establish whether some polymers were overlooked in previous studies that used sodium chloride. The detection workflow consisted of stereomicroscopy, µ-FTIR and Pyr-GC-MS which was used for simultaneous trace-level quantification of 11 environmentally relevant microplastic polymers in the water samples.

## Materials and methods

### Chemicals and reagents

The analytical workflow used methanol (*w* = 99.9%, VWR Chemicals, Oslo, Norway), dichloromethane (*w* = 99.9%, Sigma-Aldrich, France), ferrous sulphate heptahydrate (*w* = 99%) and sulphuric acid (*w* = 98%; Loba Chemie Pvt Ltd, Mumbai, India), zinc chloride (*w* = 98%, Sigma-Aldrich, Schnelldorf, Germany), absolute ethanol (*w* = 98%, VWR Chemicals, Oslo, Norway) and 30% hydrogen peroxide (Merck Specialities Pvt Ltd, Mumbai, India). The MPs calibration standard set (polymer particles dispersed in SiO_2_ and CaCO_3_; SiO_2_ and CaCO_3_ diluents, MPs-quartz wool) and polystyrene reference material (2.5 mg as thin film including methyl stearate (*w* = 5%)) were from Frontier Laboratories Ltd, Koriyama, Japan.

### Sampling

Surface water was trawled in two stratified sampling periods (wet and dry seasons, i.e. August 2023 and November 2023) from Lake Victoria (see Supplementary Information for description of the study area). The sampling was done at Ripon Falls, Katosi and Port Bell FLBs of Lake Victoria based on their fish landing systems and the nature of anthropogenic activities in their vicinity (Supplementary Information Fig. S1 and Table S1). Three parallel transects were marked within each beach, taking into consideration the effect of pollution from the shores moving into pelagic waters (bathymetric differences). At the beginning and end of each transect, non-conservable parameters of the lake water (temperature, pH, electrical conductivity, salinity, dissolved oxygen and total dissolved solids) were measured *in situ* using a portable YSI 556 MPS multiprobe meter (YSI Inc., Yellow Springs, OH, USA).

Surface water was sampled using an anodized aluminium manta trawl (Hydro-Bios Apparatebau GmbH, Altenholz, Germany) as described by Egessa et al. [8] with slight modifications. The trawl (30 cm wide, 15 cm high mouth opening, 200 cm net bag length and 0.3 mm mesh) fitted with a mechanical flowmeter and attached to a rope on a pole was operated from one side of the motorboat which kept it outside the wake zone. Along each transect, one manta trawl sample was collected (horizontally and against the water current), giving a total of 18 samples for both seasons. From the measured tow length and width of the trawl, the area (km^2^) sampled was calculated. After 20 min of continuous towing (except at Port Bell where trawling was done at 5 min intervals due to severe clogging of the soft net bucket by algae), the net was recovered and flushed using lake water to concentrate the MPs into the cod end. Samples were collected in 1 L glass bottles, covered with aluminium foil and transported to the laboratory in an ice-cooled box.

### Sample preparation

The wet peroxide oxidation protocol recommended by the National Oceanic and Atmospheric Administration for quantifying synthetic particles in water was followed [13]. Fenton reagent (20 mL of 30% hydrogen peroxide solution and 20 mL of 0.05 mol/L acidified ferrous sulphate solution) was added to the samples, and then heated on a hot plate in a flow fumehood. The temperature was maintained at 75 °C, and another 20 mL of hydrogen peroxide was added to the mixture until complete disappearance of organic matter. The resultant clear solutions were filtered through stacked 5.0-mm and 0.25-mm stainless steel sieves, and the retentate of the 0.25 mm sieve was backwashed with distilled water into a 1 L glass beaker. Thereafter, 50 mL of zinc chloride solution (700 g/L) was added and the mixture stirred for 15 min before transferring it into a density separator, which was subsequently allowed to stand for 1 h [14]. Floating MPs were filtered through glass microfiber filters (GF/C, Ø 47 mm, 1.2 µm poresize; Cytiva, China). The filter papers were transferred into petri dishes, covered loosely with aluminium foil and left to dry in air.

### Physical characteristics of the extracted MPs

Dry filters were visually examined under a ZEISS Stemi 508 stereomicroscope (Carl Zeiss Microscopy GmbH, Jena, Germany) at magnification ranging from ×6.3 to ×50. Particles were identified as MPs based on their visual characteristics (shape, distinct and uniform colour) and a break test was performed to verify their plastic nature. Micrographs of the particles were captured using a ZEISS Axiocam 208 colour camera controlled via ZEISS Labscope imaging software. The counts were standardised to particles/km^2^, particles/L and particles/m^3^, and the results from replicates were averaged for each site to yield the average abundance per site per season.

Particles were grouped into four size categories according to their longest or widest dimensions (0.3–0.9 mm, 1.0–1.9 mm, 2.0–2.9 mm, 3.0–3.9 mm and 4.0–4.9 mm) [8]. The MPs belonged to five form categories: fragments, filaments, fibres, films (planar plastic items) and pellets. In addition to sizes and forms, the MPs were sorted by their colours.

### Micro-attenuated total reflectance Fourier transform infrared spectroscopy

A randomised selection comprising six filters with microplastic particles underwent micro-attenuated total reflectance Fourier transform infrared (μATR-FTIR) spectroscopy analysis, performed using an IRTracer-100 spectrometer coupled to an AIM-9000 infrared microscope (Shimadzu Corporation, Kyoto, Japan). Background scans were performed prior to each analysis, and the spectra were collected as an average of 20 scans in the range of 4000–400 cm^−1^ at a spectral resolution of 4 cm^−1^ [8]. The polymeric composition of particles was confirmed by comparing the raw spectra to reference spectra in LabSolutions IR polymer library (version 2.2, Shimadzu Corporation, Kyoto, Japan). Matches with a hit quality index exceeding 70% were considered acceptable. For those with 60–70% similarity, individual inspection was performed in Essential FTIR (v3.5.0.225, Operant LLC, Madison, WI, USA) to confirm that sample peaks aligned with characteristic peaks of standard polymers in published literature. Matches with less than 60% agreement were excluded.

### Thermoanalytical Pyr-GC-MS

Discs were punched out of the 12 remaining filters using a 4-mm micro-puncher on a cutting mat (Frontier Laboratories Ltd, Japan) following a quarter-judgmental hybrid sampling method adapted from previous studies [10, 15]. Four discs were obtained per filter, each was fitted into a pyrolysis cup (eco-cup LF) and transferred onto an AS-1020E auto-shot sampler mounted on an EGA/PY-3030D pyrolyser (Frontier Laboratories Ltd, Japan). The pyrolysis unit was directly coupled with an Agilent 7890B GC/5977 MSD system (Agilent Technologies, Santa Clara, USA) through a split/splitless inlet. A combination of metal capillary columns (UAMP-30M-0.5F, 30 m × 0.25 mm × 0.5 μm; precolumn UAMP-2M-1.0F, 2 m × 0.25 mm × 1 μm) designed for MPs analysis was used for analytical separation.

Pyrolysis proceeded online in the single-shot mode at 600 °C for 1 min, with a pyrolyser/GC interface temperature of 300 °C. Helium was used as the carrier gas, with GC inlet temperature and pressure of 300 °C and 49 kPa (constant pressure), respectively. The column was initially held at 40 °C for 2 min, ramped up to 280 °C at 20 °C/min and then held for 10 min. Thereafter, the temperature was increased to 320 °C at 40 °C/min and maintained for 15 min. The GC was run in the split injection mode at a split ratio of 30:1. The detector was operated in the EI positive mode (70 eV, *m/z* range 29–350), while the quadrupole analyser and the ion source were at 150 °C and 230 °C, respectively. Pyrograms were acquired using Masshunter Workstation (version 10.1.49).

The filters used are produced from borosilicate glass fibres, and calibration curves for the characteristic pyrolysates of the 11 common plastics were based on the MPs-SiO_2_ standard. The MPs standard with silica as an inert diluent (0.4, 0.8, 1.2, 2.0 and 4.0 mg) was weighed on a Sartorius MCE36P-3S00-D Cubis balance (Sartorius Lab Instruments, Göttingen, Germany; max 32 mg; readability = 1 µg). Pyrograms were processed using Masshunter Unknowns Analysis, Enhanced Data Analysis and Quantitative Analysis (Agilent Technologies, Inc.). Spectral matching was done against a custom library (created using Agilent Library Editor), NIST 17 and NIST 23 libraries. Pyrolysates and their retention times were verified using F-Search All-in-One (v3.7.0, Frontier Laboratories Ltd, Japan).

### Analytical quality control and quality assurance

The entire workflow used nitrile gloves, glass, aluminium foil and stainless steel or metallic materials, and prefiltered extraction solutions to prevent contamination. Cotton laboratory coats were worn during sample preparation and analyses. Wet peroxide oxidation proceeded in a flow hood, and procedural controls were set up using prefiltered distilled water.

The Pyr-GC-MS method employed was initially validated by Frontier Laboratories Ltd using an identical pyrolyser and analytical column [16]. The injector pressure (150 kPa) and split ratio (50:1) were adapted to suit the complexity of the present samples and increase analytical sensitivity. Following Eurachem Guide [17], a partial method validation verifying the method’s performance was performed, and the detection capability under the prevailing analytical conditions was assessed.

Strictly new eco-cups were used; empty cups were pyrolysed as blanks daily. In the trial phase, plastic samples from 3D printing filaments (high-impact polystyrene, polypropylene, polylactic acid, acrylonitrile butadiene styrene, polyethylene terephthalate glycol and polyethylene), high density polyethylene barrel and a polyethylene terephthalate (PET) water bottle were pyrolysed to check instrument settings and validate polymer identification. Polystyrene reference material (as a continuing calibration-check sample according to ASTM-D8401-24 [18]), discs from the filter material, cotton buds, soda lignin, indulin AT and nitrile gloves used in handling samples were also pyrolysed. This examined the analytical selectivity of the pyrolysates and their indicator ions for potential interferences (biogenic polymers and organic materials). The limit of detection (LOD) and limit of quantification (LOQ) were calculated from the standard deviation of repeatability for the responses at 0.4 mg (*σ*) and the slope of the calibration curve (*S*) using Eqs. 1 and 2 [17, 19].

1 $$LOD=3.3\times \sigma /S$$

2 $$LOQ=10\times \sigma /S$$

### Statistical data analysis

Quantitative data on the physical characteristics of MPs was subjected to Shapiro-Wilk test of normality, and the distribution was used to decide the appropriate statistical tests. Kruskal-Wallis analysis of variance (ANOVA) or two-way ANOVA was used to establish the effects of seasons and sampling sites on the physicochemical parameters of lake water, and the abundance and characteristics of MPs in the samples. Dunn’s or Tukey’s post hoc test was then used at *P* < 0.05. Non-parametric Spearman’s rank correlation was used to explore monotonic relationships between MPs abundance and the physicochemical parameters of surface water. Principal component analysis based on standardised variables was used to explore the multivariate relationships among physicochemical parameters of surface water and MPs abundance. Statistical evaluations and data visualisation were accomplished in Origin Pro 2026 software (OriginLab Corporation, Northampton, MA 01060, USA).

For MPs quantification, a method was initially set up in quantitative analysis for GC-MS and LC-MS, and the responses were exported as CSV files into MATLAB software (version R2024b, MathWorks Inc., USA). The data was processed using a custom MATLAB script that fitted the response (peak area) against the weighed mass of the calibration samples using a linear regression model. Linearity of the calibration curves was checked using lack-of-fit *F*-test at *P* < 0.05.

## Results and discussion

### MPs are ubiquitously distributed in Lake Victoria’s surface water

During sample preparation, no additional contamination was observed. Airborne MPs during microscopic analysis were considered negligible since none of the control filters (*n* = 10) had detectable MPs. The physicochemical properties of water varied significantly between seasons and among FLBs (Kruskal-Wallis ANOVA, *P* < 0.05; see Supplementary Information Table S2 and Table S3). Although Spearman’s rank correlation test indicated that there were strong and significant positive monotonic relationships between some physicochemical parameters, only weak negative non-significant monotonic relationships existed between MPs abundance and the water quality parameters (Supplementary Information Fig. S2). This was further confirmed by principal component analysis which showed that MPs abundance did not cluster with any of the measured physicochemical parameters ( Supplementary Information Fig. S3).

All the 18 trawl samples had MPs, with a total of 191 putative particles detected (Supplementary Information Table S4). The lowest and highest mean abundances of MPs (39 × 10^3^ ± 21 × 10^3^ particles/km^2^ or 0.13 ± 0.06 particles/m^3^ and 87 × 10^3^ ± 23 × 10^3^ particles/km^2^ or 0.61 ± 0.38 particles/m^3^) were items in the wet-season and dry-season samples from Ripon Falls and Katosi FLBs, respectively. In the dry season, the trend of MPs abundance (particles/km^2^) was Katosi (87 × 10^3^ ± 23 × 10^3^) > Ripon Falls (59 × 10^3^ ± 13 × 10^3^) > Port Bell (45 × 10^3^ ± 23 × 10^3^). The abundance of MPs in particles/m^3^ was Katosi (0.61 ± 0.38) > Port Bell (0.60 ± 0.48) > Ripon Falls (0.47 ± 0.25). In the wet season, the abundances in particles/km^2^ and particles/m^3^ were Port Bell (61 × 10^3^ ± 12 × 10^3^) > Katosi (55 × 10^3^ ± 18 × 10^3^) > Ripon Falls (40 × 10^3^ ± 21 × 10^3^), and Katosi (0.44 ± 0.06) > Port Bell (0.43 ± 0.10) > Ripon Falls (0.13 ± 0.06), respectively. Seasons and sampling location had negligible effects on the abundance of MPs (two-way ANOVA, *P* = 0.70).

The ubiquity of MPs in water from all the transects is in agreement with previous microplastic studies of Lake Victoria [8, 20]. The highest MPs abundance observed at Katosi may be due to the high level of both fishing and commercial activities in this otherwise rural setting. The slightly higher MPs abundance at Port Bell during the wet season could arise from industrial wastes and stormwater entering the inner Murchison Bay through the Nakivubo channel [20–22]. The levels of MPs found in the present study are lower than 2.8 × 10^3^–329.2 × 10^3^ particles/km^2^ (0.02–2.19 particles/m^3^), 688 particles/m^3^ and 0.25–3.33 particles/m^3^ as previously quantified in some Lake Victoria bays [8, 20, 21]. This could be due to the highly varied use of Lake Victoria, which results in different environmental pressures at the studied portions of the lake. The abundances were also lower than some of the highest pelagic MPs pollution to date, such as 230 × 10^3^ particles/km^2^ in Lake Ontario of the USA [23] and 5 × 10^3^–758 × 10^3^ particles/km^2^ in the Chinese Qinghai Lake [24]. Lower or comparable abundance of MPs has been reported in Lake Erie, USA (45 × 10^3^ particles/km^2^) [23]; Luruaco Lake, Columbia (0–3.83 particles/L) [25]; Lake Naivasha, Kenya (0.41 particles/m^3^) [26]; and Italian lakes: Iseo, Maggiore and Garda (25 × 10^3^–40 × 10^3^ particles/km^2^) [27].

### Fragments are the dominant forms of MPs in Lake Victoria surface water

**Fig. 1:**
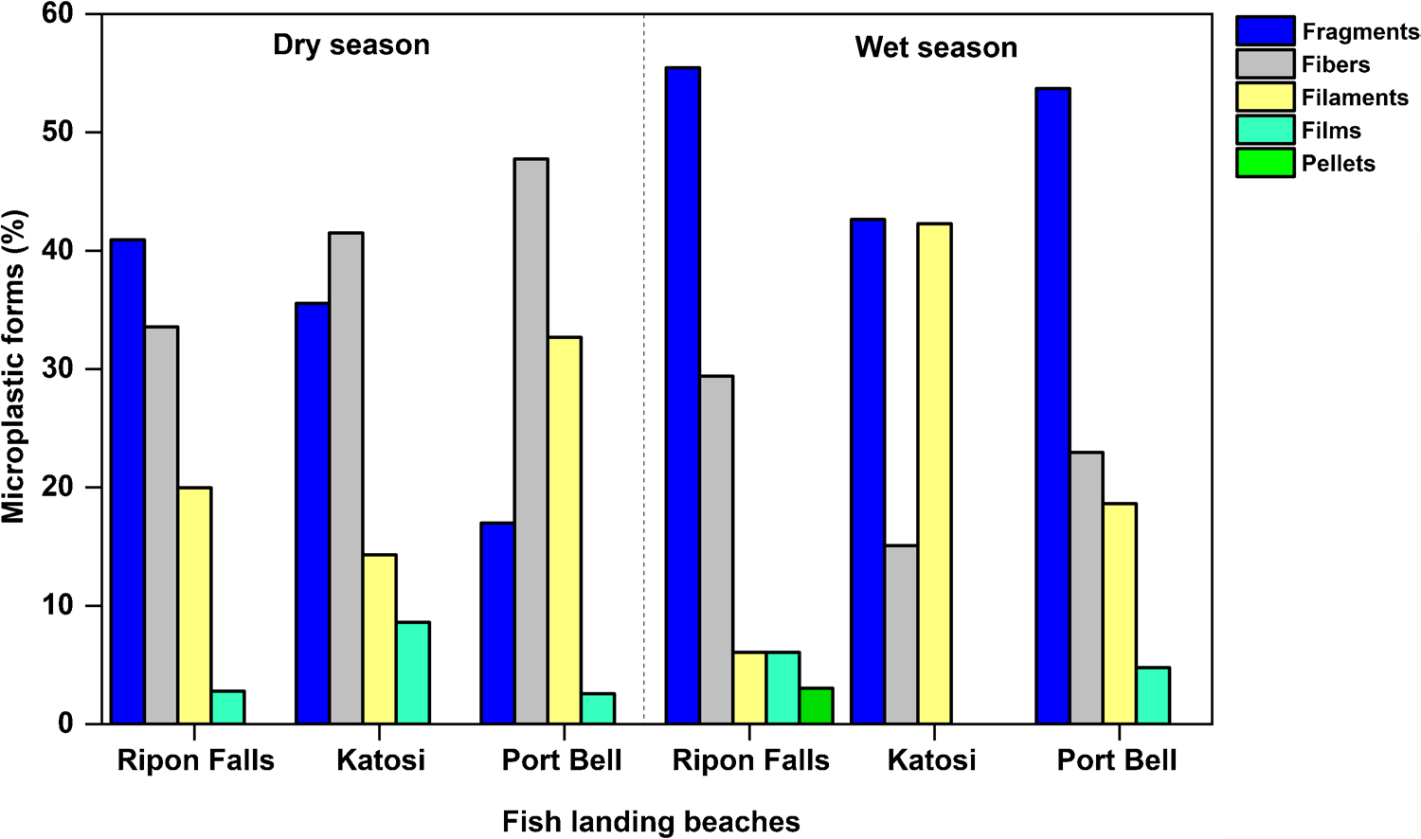
Forms of microplastics identified in surface water from Lake Victoria

Fragments had 40.9% average contribution to the total microplastic forms detected. This was followed by fibres (31.7%), filaments (22.1%), films (4.1%) and pellets (0.5%). An exception to this trend were samples trawled from Port Bell during the dry season which had mostly fibres (47.8%) and filaments (32.7%) (Fig. 1, Supplementary Information Fig. S4). Pellets (9.1%) were only recorded in wet-season samples from Ripon Falls, and this is the first time this form of MPs was observed in Lake Victoria. Statistically, seasons and sampling location had insignificant influence on the forms of MPs in the samples as per Kruskal-Wallis test (*H* = 17.0, *P* > 0.05).

These results are consistent with Egessa et al. [8] and Kakooza [21], who profiled fragments and fibres as the most abundant forms of MPs in surface water of Lake Victoria. These proportions of MPs categories are comparable to most lacustrine MPs data, such as those from Red Hills Lake [28], Lake Phewa [29], Lake Erie, Lake Ontario [23], Luruaco Lake [25] and Tocagua Lake [30], where fragments and fibres were always dominant in surface water.

### Blue microparticles were the most frequent

The particles could be categorised into nine colours, and blue was the prominent colour (47.1–70.6%) in the dry season (Supplementary Information Fig. S5). During the wet season, blue (40.3%) and black (15.2%) particles were frequently observed at Port Bell. Transparent (19.4%) and black (19.0%), and black (42.7%) and blue (21.4%) were the colours of particles from Katosi and Ripon Falls, respectively. Kruskal-Wallis ANOVA revealed that microplastic colours did not significantly differ among FLBs and between seasons (*H* = 5.09, *P* = 0.83). Studies of Lake Victoria indicated that white/transparent and blue were the primary colours of MPs in water, although green, black, yellow, purple and red particles also occurred in lower proportions [8, 21]. In lakes such as Phewa of Nepal [29], Towuti of Indonesia [31] and Laguna of Philippines [32], blue and transparent MPs were the most prominent.

The dominance of blue plastic debris is linked to the fact that blue is the preferred colour for synthetic plastic materials worldwide. Blue phthalocyanine pigments are by far the most photostable dyes commonly used in plastics, which could explain their persistence and prominence in the environment, and subsequent higher visual detection during stereomicroscopic analysis [33]. Barrels and plastic bottle caps for packaged drinking water in Uganda are mostly blue, and these bottles are used as floats for fishnets in Lake Victoria [8, 34]. The bottles were sited during sampling, and under intense hydrodynamic stress, they can fragment into MPs. It is also discussed that white and transparent MPs can result from photodegradation and colour fading of originally pigmented plastics after prolonged exposure to intense ultraviolet radiation [35]. Lake Victoria straddles the equator, and its northern shores in Uganda that were sampled receive high solar radiation during the dry season, which could enhance photodegradation of macroplastics into MPs.

### Heteroaggregation favour the dominance of large-sized MPs

Particles in the size groups of 4.0–4.9 mm (9.1–54.1%), 3.0–3.9 mm (7.8–41.8%) and 2.0–2.9 mm (12.1–29.0%) constituted the highest total percentage of the detected MPs. Port Bell samples had no detectable MPs in the size ranges of 0.3–0.9 mm and 1.0–1.9 mm during the wet and dry seasons, respectively (Supplementary Information Fig. S6). Spatiotemporally, wet-season surface water from Port Bell had the highest proportion of MPs measuring 4.0–4.9 mm (54.1%), followed by samples from Ripon Falls trawled in the dry season (26.7%), and then dry-season samples from Port Bell (26.3%). There were significant differences in the sizes of MPs among the FLBs and between seasons (*H* = 17.0, *P* = 0.002).

Earlier studies found that the abundance of MPs in Lake Victoria tended to decrease with increasing sizes, as it generally does [8, 21], but the present data do not support that trend. Particle-based concentration of MPs in surface water is expected to inversely correlate with their sizes (follow a power law) due to fragmentation and other dispersal dynamics [36]. In the current study, the size variations could reflect the uniqueness of the seasons and FLBs, but may have also been potentially introduced by the sampling and sample preparation methods used. For example, zinc chloride solution was preferred for salinity-based density separation in the present study, while previous studies [8, 20, 21] used sodium chloride solution, which has a lower density. Spatial MPs size distribution patterns have also been associated with differences in plastic input, hydrodynamic conditions and biofouling [24]. Heteroaggregation of small-sized MPs with algae and organic matter, which was observed for Port Bell samples, can lead to vertical transport of such MPs from surface water to the lake bottom [37].

### Polyethylene and polypropylene were the dominating polymer types according to µ-FTIR

Using µ-FTIR spectroscopy, 48.1% (25 of the 52 particles) were found to be MPs belonging to five polymer groups. The majority of the particles in the dry-season samples were made of polypropylene (PP; Supplementary Information Table S5). Across the FLBs, polyethylene (PE) constituted 36% of the recovered plastic debris. In the wet-season samples, PE, PE/PP copolymer and polyacrylamide were the major polymers in samples from the three transects at Katosi, Port Bell and Ripon Falls. Spatiotemporally, PP was dominant during the dry season, while the wet season was dominated by PE and polyacrylamide. Two particles in the dry-season and wet-season samples from Ripon Falls were found to be made of polyamide (nylon 6) and PE/PP copolymer. Taken together, PE was the most prevalent polymer, followed by PP and then polyacrylamide.

The infrared spectral features of particles identified to contain PE (Supplementary Information Fig. S7) had characteristic wavenumbers associated with CH_2_ rocking (717 cm^−1^), CH_2_ bending (1471 cm^−1^) and CH stretching (2846 and 2916 cm^−1^). Wavenumbers attributed to CH_3_ bending, CH_2_ bending and CH stretching (1377 cm^−1^, 1454 cm^−1^ and 2914/2954 cm^−1^) were evident in the spectra of particles confirmed to contain PP [38].

These results align well with previous studies of Lake Victoria waters, in which PE and PP were the dominant microplastic polymers [8, 21]. This is also in agreement with studies of Laguna Lake, Philippines [32], Gulshan and Hatir Jheel lakes of Bangladesh [39] and Lake Kallavesi of Finland [40]. The prevalence of PE and PP fragments in this study suggests that they stem from the breakdown of macroplastics that ended up in the lake. Water bottle caps, straws, most household items, single-use grocery and garbage bags are usually produced from PP and PE [8]. Some of these items were evident at the FLBs during the sampling campaigns.

### Polymer identification improved through appropriate choice of diagnostic pyrolysates

Pyr-GC-MS is an indirect analytical technique that thermally degrades the target polymers and analyses their specific pyrolysates. The most abundant pyrolysis product of a polymer is often, but not always, the best marker that can be used for quantitative analysis. Thus, the selection of specific indicator (diagnostic) compounds and ions is important. The pyrolysates used in the present study were required to meet certain criteria: they had to (i) be detectable in the pyrograms at all calibration weights and within ±1 min retention time window, (ii) demonstrate high pyrolytic yields in the calibration samples and (iii) exhibit sufficient specificity for analytical quantification of the respective polymer, as confirmed by previous Pyr-GC-MS studies [9, 11, 19, 41, 42]. For polymer identification (qualification), other tracers were also monitored (Table 1).

**Table 1:** Pyrolysates used for identification and quantification of microplastics using MPs-SiO_2_ standard

Polymers	Characteristic pyrolysis products^a^	Abbreviation	Indicator ions (*m/z*)^b^	*t*_R_ (min)
Poly(ethylene terephthalate) (PET)	Benzoic acid	BA	***105***, 77, 122	12.910
	Benzophenone	BP	105, 77, 182	17.368
Nylon 6 (N6)	ε-Caprolactam	Capro	**85**, 63, *113*	14.451
Nylon 66 (N66)	Cyclopentanone	CP	***84****,* 55, 56	8.764
Acrylonitrile-butadiene styrene (ABS; terpolymer)	2-Phenethyl-4-phenylpent-4-enenitrile	SAS	***170***, 91,** 115**	23.011
	3-Phenylpent-2-enenitrile	–	91, 77, 157	15.564
	4-Phenylbutanenitrile	–	91, 77, 145	14.866
Styrene butadiene rubber (SBR; copolymer)	4-Phenylcyclohexene (*SB hybrid dimer*)	4-PCH	***104***, 91, 158	14.626
	4-Vinylcyclohexene (*butadiene dimer*)	4-VCH	**108**, 54, 79	8.946
Poly(methyl methacrylate) (PMMA)	Methyl methacrylate	MMA	**69**, 99, *100*	7.248
Polycarbonate (PC)	Bisphenol A	BPA	***213***, 119, 228	23.237
	4-Isopropenylphenol	IPP	134, 91, 119	14.222
Poly(vinyl chloride)(PVC)	Naphthalene	Nap	***128****,* 63, 115	13.489
	1,2-Dihydronaphthalene	–	115, 129, 130	13.192
Polyethylene (PE)	1,20-Heneicosadiene	C21″	***82***, 95, 292	20.948
	1-Undecene	C11′	55, 97, 111, 154	11.622
Polypropylene (PP)	2,4-Dimethyl-1-heptene (*propylene trimer*)	C9′	**55**, 70, *126*	8.908
	2,3,3-Trimethyl-4-nonene (*propylene tetramer*)	C12′	55, 111, 168	20.568
Polystyrene (PS)	1,3-Diphenylbut-3-ene (*styrene dimer*)	SS	**91**, 208, 130	17.805
	Styrene (*monomer*)	S	**104**, 77, 78	10.098
	α-Methylstyrene	AMS	118, 103, 117	11.009

^a^The first listed pyrolysis product of each polymer was used for quantitative analysis

^b^Ions used for integration, in order of their entry; in **bold** are primary quantitation ions, based on their intensities; in *italics* are recommended ions by Frontier Laboratories Ltd for MPs quantification using MPs-SiO_2_ standard

**Fig. 2:**
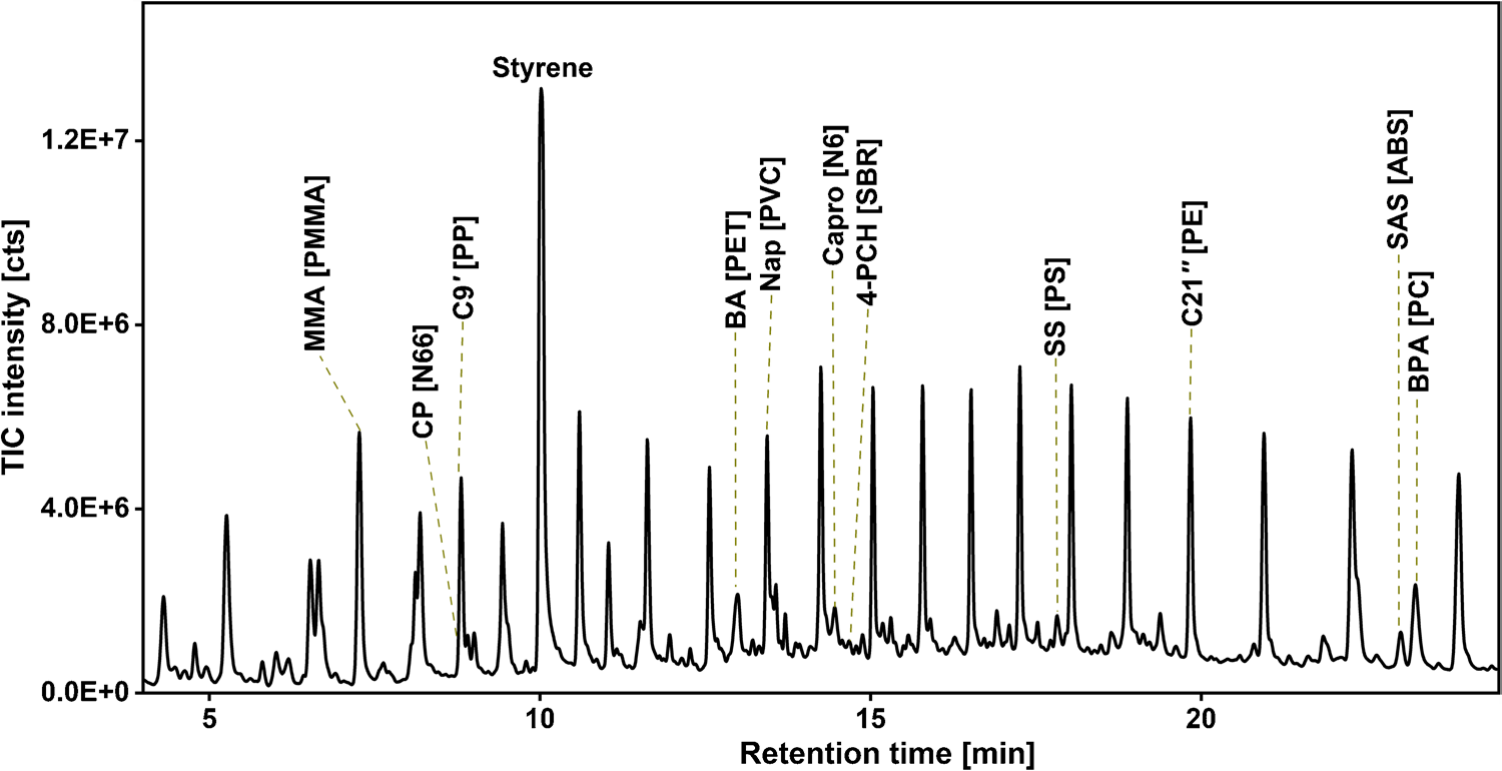
Pyrogram of MPs-SiO_2_ standard (4.0 mg). Labels indicate index compounds and their polymers (in square brackets, abbreviations defined in Table 1). Retention time was locked to styrene

The pyrolysis of PE produced a complex mixture of hydrocarbons characterised by alkanes, α-alkenes and α,ω-alkenes of various chain lengths. The C21-α,ω-alkadiene (1,20-heneicosadiene; C21″) was chosen for quantification (Fig. 2) while 1-undecene (C11′) was used as a PE tracer. Hexadec-1-ene (C16′), 1,11-dodecadiene (C12″) and 1,19-eicosadiene (C20″), which are known PE tracers, were only detected in the water-sample pyrograms and could not be monitored using the MPs-SiO_2_ calibration. The characteristic pyrolysates of PP were branched alkenes, with propylene trimer (2,4-dimethyl-1-heptene; C9′) as the main product. The tetramer of propylene (2,3,3-trimethyl-4-nonene) was used as a qualifier.

For polystyrene (PS), the styrene dimer (1,3-diphenylbut-3-ene), styrene and α-methyl styrene were the primary detected pyrolysis products. Styrene trimers (2,4,6-triphenylhex-1-ene and 1,3,5-triphenylcyclohexane) were detected in the 0.8 mg calibration and water samples. The styrene monomer was the most abundant compound in the calibration samples. However, it is a less suitable marker since its production from pyrolytic breakdown of other styrenic polymers and natural materials is well known [9, 11]. It is recommended to use a representative pyrolysate from either the dimer or trimer groups, as their generation during pyrolysis would unambiguously confirm the presence of PS in environmental matrices [42, 43]. Since the trimer was not consistently detected across all the calibration samples, the styrene dimer (1,3-diphenylbut-3-ene) was preferentially used, as it is another widely recognised oligomer for PS quantification [15, 42, 43].

**Fig. 3:**
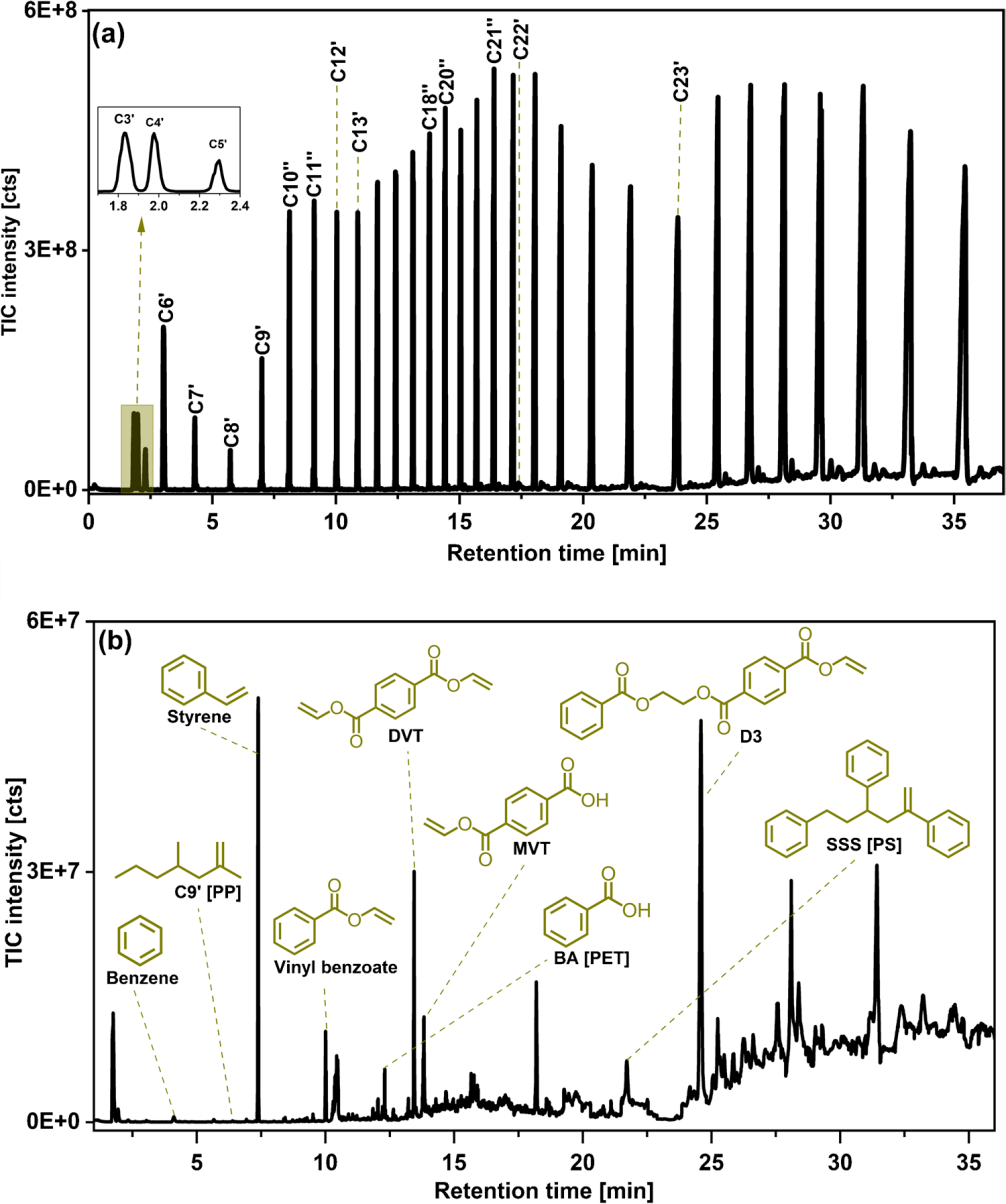
Pyrograms of pyrolysed method validation materials. **a** High-density polyethylene barrel: C3′ (propylene), C4′ (1-butene), C5′ (1-pentene), C7′ (1-heptene), C8′ (1-octene), C9′ (1-nonene), C10″ (1,9-decadiene), C11″ (1,10-undecadiene), C12″ (1,11-dodecadiene), C13′ (6-tridecene), C18″ (1,17-octadecadiene), C20″ (1,19-eicosadiene), C21″ (1,20-heneicosadiene), C22′ (1-docosene) and C23′ (1-tricosene). **b** PET water bottle: C9′, 2,4-dimethyl-1-heptene; BA, benzoic acid; DVT, divinyl terephthalate; MVT, monovinyl terephthalate; D3, 2-(benzoyloxy)ethyl vinyl terephthalate; and SSS, styrene trimer (2,4,6-triphenylhex-1-ene)

The hybrid trimer 2-phenethyl-4-phenylpent-4-enenitrile from the acrylonitrile-butadiene-styrene terpolymer was detected. It satisfies the fundamental requirement of specificity, but it had very low pyrolytic yields in the water samples. In complex environmental matrices, pyrolysate signals are expected to be masked or suppressed due to co-eluting compounds, organic matter or matrix effects, and pyrolysate specificity should always take precedence over its abundance [44]. For poly(methyl methacrylate) (PMMA), poly(vinyl chloride) (PVC), PET, polycarbonate, nylon 6 (N6) and nylon 66 (N66), the definitively assignable pyrolysis products as per previous Pyr-GC-MS studies of MPs were used [19, 45]. Since matrix effects are unavoidable in the pyrolysis of samples containing PE, PS and PVC, the use of multiple diagnostic compounds for each polymer, whenever possible, is recommended to minimise the risk of false positives [11]. The presence of PE, for example, is confirmed whenever one or more homologous series of triplets in the C_7_-C_41_ range is present (Fig. 3), and one or two compounds from the series may be selected for quantification [15]. For this reason, pyrolysis products such as 2,3,3-trimethyl-4-nonene, 3-phenylpent-2-enenitrile, 4-vinylcyclohexene, 4-isopropenylphenol, 4-phenylbutanenitrile, benzophenone and 1,2-dihydronaphthalene were exclusively monitored as polymer qualifiers (Table 1).

### The validated Pyr-GC-MS method was used for polymer quantification

The partial method validation entailed evaluating parameters, such as analytical selectivity of pyrolysis products, linearity, LOD and LOQ, in addition to other quality control/quality assurance measures. Nitrile gloves and blanks (empty pyrolysis cups) showed no contribution to the analytes’ responses (Supplementary Information Table S6 and Fig. S8). The filter material and phthalate-free quartz wool had false positive responses of bisphenol A and ɛ-caprolactam, but the wool was not used to cover any of the samples pyrolysed. Soda lignin and indulin (a kraft lignin), analysed as prominent potential interferences, contributed to the responses of some pyrolysates, a fact that is consistent with published literature data [9, 11].

The non-specificity of some pyrolysates and the complex nature of environmental samples were evident from the results. For instance, ABS and high-impact polystyrene 3D-printing filaments showed traces of PMMA, PE and PET, in addition to high concentrations of ABS, PS, PP and PVC. Using benzoic acid as the diagnostic pyrolysate, a water bottle showed a lower concentration of PET. However, other PET markers, such as vinyl benzoate, divinyl terephthalate, monovinyl terephthalate and 2-(benzoyloxy)ethyl vinyl terephthalate, were present in the pyrogram (Fig. 3). In contrast, these PET tracers were not detected in the pyrograms of the calibration and water samples, which could be because they contained inorganic matrices known to reduce the peak areas of these pyrolysates [12].

Five-point external calibration curves for all the polymers (0.35–193.14 µg) showed acceptable linearity (*R*^2^ ≥ 0.975; Table 2, Supplementary Information Fig. S9). The LOD and LOQ were in the ranges of 0.01–14.71 µg and 0.03–49.06 µg, respectively. Polycarbonate, PVC, PET and N6 had the lowest LOD values when compared to previous reports, while the rest of the polymers had LOD values that were at least thrice those previously determined for Pyr-GC-MS analysis of surface water or the MPs-SiO_2_ standard [16, 42, 43]. This may be attributed to the different generation efficiencies of the pyrolysates from microplastic polymers, or the use of undissolved calibration standards. The LOD and LOQ of PE were the highest because the pyrolysis of this homopolymer produces hydrocarbons with discrete chain lengths which are very broadly distributed. Conversely, pyrolysis of N6 yields ɛ-caprolactam as the major pyrolysate, and in such a case, lower LOD and LOQ are expected [46].

**Table 2:** Linearity, limit of detection (LOD) and limit of quantification (LOQ) of microplastic polymers quantified by Pyr-GC-MS

Polymer	Pyrolysates	Linear range (µg)	Linearity (*R*^2^)	LOD (µg)	LOQ (µg)	LOD (µg)^a^
PET	Benzoic acid	1.79–17.91	0.984	0.24	0.81	0.94
N6	ɛ-Caprolactam	0.70–6.50	0.995	0.15	0.52	0.05
N66	Cyclopentanone	1.90–19.01	0.981	1.13	3.77	0.32
ABS	2-Phenethyl-4-phenylpent-4-enenitrile	2.03–20.32	0.983	1.78	5.95	0.05
SBR	4-Phenylcyclohexene	2.10–21.02	0.981	0.92	3.08	0.15
PMMA	Methyl methacrylate	1.38–13.81	0.997	0.30	1.01	0.03
PC	Bisphenol A	0.35–3.50	0.977	0.01	0.03	0.24
PVC	Naphthalene	5.60–52.14	0.998	0.32	1.09	0.38
PE	1,20-Heneicosadiene	19.30–193.14	0.987	14.71	49.06	1.90
PP	2,4-Dimethyl-1-heptene	4.82–48.24	0.982	4.24	14.13	0.56
PS	1,3-Diphenylbut-3-ene	1.25–12.51	0.975	0.34	1.16	0.11 ^b^

^a^Technical note: PYA1-145E [16]; ^b^For styrene trimer: 2,4,6-triphenyl-1-hexene

### Quantitative Pyr-GC-MS confirmed and complemented µ-FTIR results

**Fig. 4:**
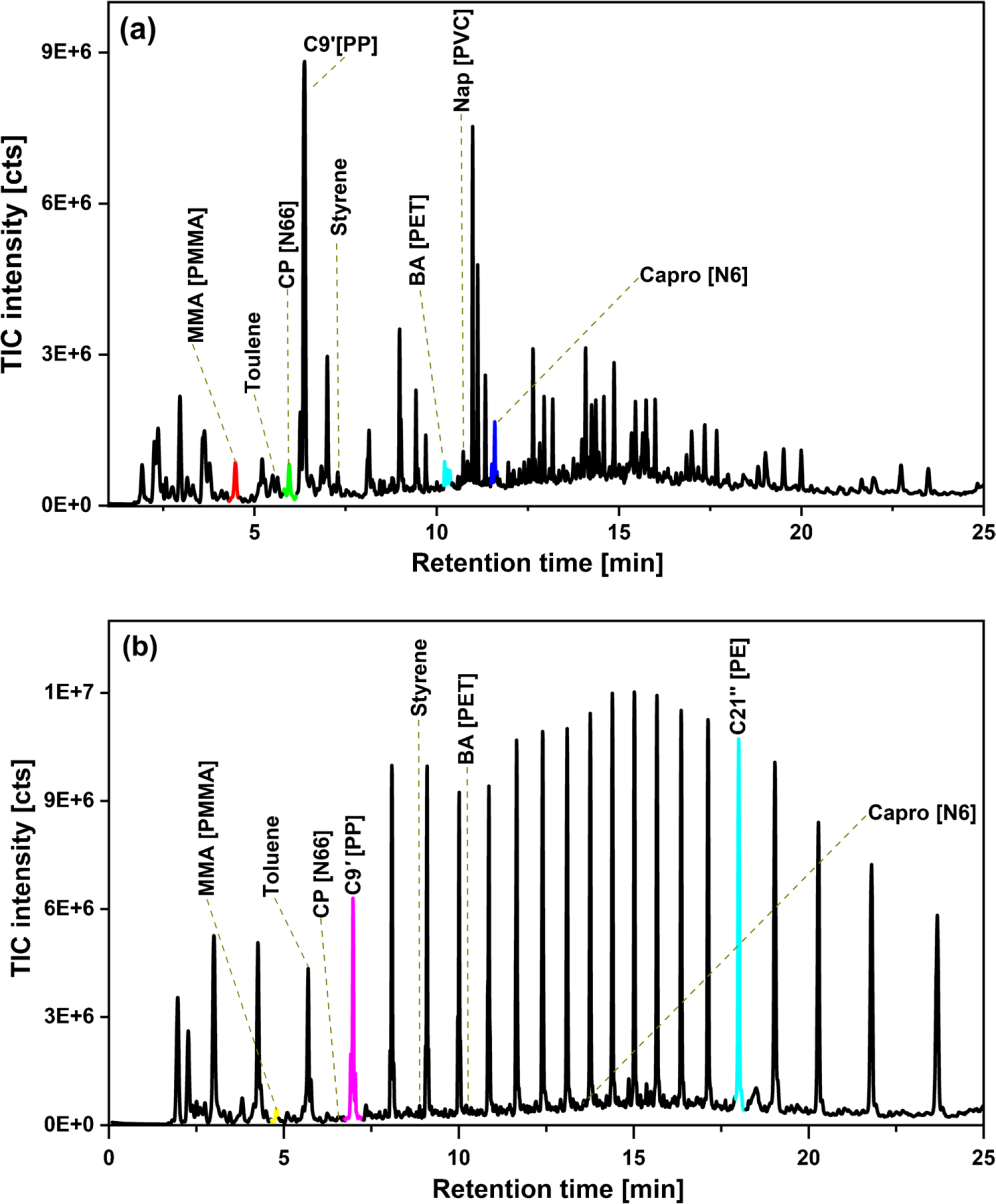
Pyrograms of microplastics in surface water from Lake Victoria. **a** Sample with poly(methyl methacrylate) (Port Bell 01). **b** Most contaminated sample (Port Bell 03). Labels are defined in Table 1. Characteristic pyrolysis products of major polymers above their LOQ are coloured

In agreement with previous µ-FTIR results, Pyr-GC-MS detected and quantified PE (0.058–0.34 µg/L), PP (0.024 µg/L and 0.043 µg/L) and N6 (0.0051–0.064 µg/L) in the samples (Table 3; Supplementary Information Table S5). The MPs quantified ranged from two to five different polymers in one sample. Samples from Port Bell were the most contaminated (Fig. 4), followed by those from Katosi and Ripon Falls. This result agreed with expectations since samples from Port Bell were dominated by large-sized MPs (3.0–4.9 mm). Port Bell is both a fish landing beach and a freight port, and receives wastes from Kampala City (through the Nakivubo channel that drains into the inner Murchison Bay) [22] as well as from the operational industries in Luzira Industrial Park.

**Table 3:** Concentration of microplastics (µg/L) in surface water of Lake Victoria

**Polymer**^*a*^	**Dry season (August 2023)**						**Wet season (November 2023)**					
	**Ripon Falls 01**	**Ripon Falls 02**	**Katosi 01**	**Katosi 02**	**Port Bell 01**	**Port Bell 02**	**Ripon Falls 03**	**Ripon Falls 04**	**Katosi 03**	**Katosi 04**	**Port Bell 03**	**Port Bell 04**
PET	0.0075	0.0095	0.027	0.0087	0.016	0.017	0.0030	0.0029	0.011	0.0082	0.0093	0.011
N6	0.0093	0.016	0.0082	0.0091	0.022	0.029	0.0051	0.0056	0.034	0.013	0.064	0.011
N66	0.0089	0.015	< LOD	0.0029	0.011	0.024	0.0022	0.0022	0.037	0.014	0.084	< LOQ
PE	0.058	< LOD	< LOD	< LOD	< LOD	< LOQ	< LOQ	< LOD	< LOD	0.14	0.34	< LOD
PP	< LOD	< LOQ	< LOD	< LOD	< LOQ	< LOQ	< LOQ	< LOD	< LOD	0.043	0.024	< LOQ
PMMA	< LOD	< LOD	< LOD	< LOD	0.0036	< LOD	< LOD	< LOD	< LOQ	< LOD	< LOQ	< LOD

^a^Polymer concentrations were normalised to the volume of water filtered. The LOD and LOQ are provided in Table 2

Wet-season samples had higher polymer concentrations, with N66 (0.0022–0.084 µg/L), PET (0.0029–0.027 µg/L) and PMMA (0.0036 µg/L) detected and quantified for the first time in Lake Victoria waters. Only N6 and PET were detected and quantified across all the FLBs in both seasons. Although at concentrations lower than their LOQ, PE, PP and PMMA were also detected, especially in Port Bell samples. The presence of quantifiable PMMA in only one sample from Port Bell suggests that it could be from the abrasion of structural plastics, protective coatings and paints since there are active shipping lanes at this port using roll-on/roll-off freight vessels. A previous study in the North Sea [47] linked elevated PMMA levels in water samples to intense shipping traffic and the associated ship materials, such as coatings and structural plastics, which could easily be the case also at Port Bell.

Polystyrene, ABS, SBR and PVC were below LOD in all the samples. In the context of polystyrene, this could hold true as only traces or undetectable levels of styrene monomer were present in even the most contaminated samples. For PVC, traces of its diagnostic pyrolysate were present in some samples (see Fig. 4). This apparent suppression of PVC arises from the influence of incorporated functional additives during its production or its susceptibility to photodegradation and weathering which deteriorate the polymer’s spectral features [48]. Quantitative Pyr-GC-MS analysis of PVC targeting naphthalene and its derivatives has also been a subject of analytical scrutiny because these markers are at the same time possible pyrolysis products of natural organic matter (Supplementary Information Table S6 illustrates this for the case of soda lignin). Wet peroxide oxidation does not seem to completely eliminate such materials that generate those pyrolysis interferences, especially in organic-rich matrices. As a consequence, few studies have unequivocally quantified PVC using naphthalene and its derivatives [10], and in most cases limited coverage is provided for this polymer. So far, the analytical concerns point to the perceived low specificity and reduced sensitivity of the markers [15, 49] or the non-linearity of their responses relative to the expected polymer concentrations in the calibration samples [50]. Polycarbonate was quantified in all the samples well above the LOQ of 0.03 µg. As mentioned above, the filter material contributed to the responses of bisphenol A, and blank correction resulted in negative concentrations in some cases. Consequently, polycarbonate was omitted from the target list of the polymers quantified.

Polyethylene and PP were the most frequently identified polymers by µ-FTIR and were quantified at the highest concentrations by Pyr-GC-MS. However, they were quantified in fewer samples. This could be because in complex matrices, the broad pyrolysate peaks of PE and PP get masked, complicating trace-level quantification. The use of C21-α,ω-alkadiene as a marker for PE quantification in complex matrices is recommended for improved selectivity [11, 51] but it gives a relatively higher LOQ for PE compared with other alkenes in the homologous series, resulting in few quantifiable samples. Conversely, PET, N6 and N66 were quantified most reliably in the samples, despite being detected less by µ-FTIR. These polymers produce highly specific marker compounds during pyrolysis, which can improve their quantification [46]. The results obtained in the present study showed that µ-FTIR provides accurate polymer identification for the most abundant MPs whereas Pyr-GC-MS contributes mass-based concentration and chemical confirmation of polymers that may not be detected by µ-FTIR. Together, µ-FTIR and Pyr-GC-MS provided a more comprehensive characterisation of MPs and their spatiotemporal dynamics in the eutrophic waters of Lake Victoria.

With regard to the quantified microplastic polymers in Lake Victoria surface water, there exist only a handful of relevant studies of lacustrine ecosystems that have used Pyr-GC-MS for simultaneous identification or quantification of all the plastic polymers investigated. Perhaps the best comparison can be made to Lake Superior (USA), the only freshwater lake exceeding the surface area of Lake Victoria. Qualitative Pyr-GC-MS analysis of water from Lake Superior detected PVC, PP, PE and PET [52, 53]. Elsewhere, Gomiero et al. [42] quantified PE, PP, PS, PET and polyamide in water filtered from Langevatn Lake (Norway) with a combined concentration of 0.093 µg/L. In Lake Vombsjön (Sweden), PP, PS, PVC, polyamide and polyester were quantified in the range of 0.00014–0.0054 µg/L [43]. A recent study in Taihu Lake (China) quantified 1–100 μm sized MPs (PE, PP, PET, polystyrene and PMMA) at concentrations of 229 to 1182 μg/L [54]. All these concentrations, except for MPs in Taihu Lake, are lower than those of the six polymers quantified in the Lake Victoria samples of the present study.

## Conclusions

This study was the first of its kind to assess the spatiotemporal dynamics of MPs in the East-African waterbody of Lake Victoria, complementing particle-based analytical techniques with quantitative Pyr-GC-MS. All the trawl samples using a 0.3-mm manta net contained MPs, but the particle-based levels were lower than previously detected in other stretches of the lake. A total of 191 particles, mostly blue fragments and fibres measuring 0.3–4.9 mm in sizes, were detected. The lowest and highest mean abundances of MPs (0.13 ± 0.06 particles/m^3^ and 0.61 ± 0.38 particles/m^3^) were obtained in the wet-season and dry-season samples from Ripon Falls and Katosi FLBs, respectively. Seasons and sampling location had negligible effects on the abundance of MPs.

For identification of the most abundant polymers (PE, PP and N6), μ-FTIR was well suited with minimal matrix interference. In contrast, Pyr-GC-MS using highly specific and multiple diagnostic pyrolysates increased confidence in polymer identification and enabled both detection and quantification of seven polymers (PE, PP, N6, N66, PMMA and PET), including those that were not identified by μ-FTIR. Accordingly, sequential μ-FTIR and Pyr-GC-MS enabled comprehensive analysis of MPs in eutrophic surface waters, overcoming the inherent limitations of each technique and emphasising the need to embrace a ‘not-one-size-fits-all’ analytical strategy for MPs analysis. Future analytical workflows could benefit from integrating thermal extraction-desorption GC-MS prior to pyrolysis and installing a backflush to reduce the total analysis time, improve performance and extend the Pyr-GC-MS method to other complex environmental matrices.

## Supplementary Information

Below is the link to the electronic supplementary material.

Supplementary file1 (PDF 1.02 MB)

## Data Availability

Relevant data supporting the conclusions of this study are within this article and its supplementary files.
