# Supplementary material for: Not one-size-fits-all: µ-FTIR and pyrolysis GC-MS for complementary analysis of microplastics in eutrophic surface water
Source: Anal Bioanal Chem. 2026 Mar 22;418(16):5233–46. doi: 10.1007/s00216-026-06446-w (PMC13423919; doi:10.1007/s00216-026-06446-w)
Supplement: Supplementary file 1 — Supplementary file1 (PDF 1.02 MB) [file 216_2026_6446_MOESM1_ESM.pdf]

Supporting Information

Not one-size-fits-all: µ-FTIR and pyrolysis GC-MS for complementary analysis of microplastics in eutrophic surface water

Timothy Omara [a,b], Barbora Benetková [a], Ivan Sumerskii [c], Patrick Ssebugere [b], Christine Kyarimpa [d], Solomon Omwoma Lugasi [e], Thomas Rosenau [a,f], Christine Betty Nagawa [g], Stefan Böhmdorfer [a*]

[a] Institute of Chemistry of Renewable Resources, Department of Natural Sciences and Sustainable Resources, BOKU University, Konrad-Lorenz-Straße 24, 3430 Tulln, Austria.

[b] Department of Chemistry, College of Natural Sciences, Makerere University, P.O. Box 7062, Kampala, Uganda.

[c] Core Facility Analysis of Lignocellulosics (ALICE), BOKU University, Konrad-Lorenz-Straße 24, 3430 Tulln, Austria.

[d] Department of Chemistry, Faculty of Science, Kyambogo University, P.O. Box 1, Kampala, Uganda.

[e] Department of Physical Sciences, Jaramogi Oginga Odinga University of Science and Technology, P.O. Box 210-40601, Bondo, Kenya.

[f] Johan Gadolin Process Chemistry Centre, Åbo Akademi University, Porthansgatan 3, 20500 Åbo/Turku, Finland.

[g] Department of Forestry, Biodiversity and Nature Conservation, College of Agricultural and Environmental Sciences, Makerere University, P.O. Box 7062, Kampala, Uganda.

*Corresponding author: stefan.boehmdorfer@boku.ac.at

*This **Supporting Information** has detailed introduction to the study area, the particle counts, colours, forms, size categories, and polymer composition of microplastic particles identified, and water quality parameters of Lake Victoria. The quality assurance materials pyrolysed, their representative pyrograms and the calibration curves of the eleven target polymers are also presented. It has 24 pages consisting of 6 tables and 9 figures.*

S1. Study area description

Lake Victoria (L. Victoria) is a freshwater body with the largest export-oriented inland fishery in the world [1]. Also called Victoria Nyanza, Ukerewe or Nalubaale, L. Victoria is the largest exoreic lake in Africa that lies between the Eastern and Western Great Rift Valleys (Fig. S1). It is the second largest freshwater lake in the world (after Lake Superior), the largest tropical lake known [2, 3] and the source of River Nile, which is the longest river in Africa. The lake touches the equator on its northern part which lies mostly in Uganda [4]. Lake Victoria is also known for having many indenting archipelagos constituted by 985 isles, 84 of which constitute the Ugandan Ssese chain.

There are more than 100 fish landing beaches (FLBs) on L. Victoria. On the Ugandan side, some of the well-known FLBs include Kasensero and Kyabasimba in Rakai District, Kasenyi and Kigungu in Wakiso District, Katosi and Ssenyi in Mukono District, Masese, Ripon Falls and Wairaka in Jinja District, and Port Bell and Ggaba in Kampala District [5]. This study considered Ripon Falls, Katosi and Port Bell (Table S1).

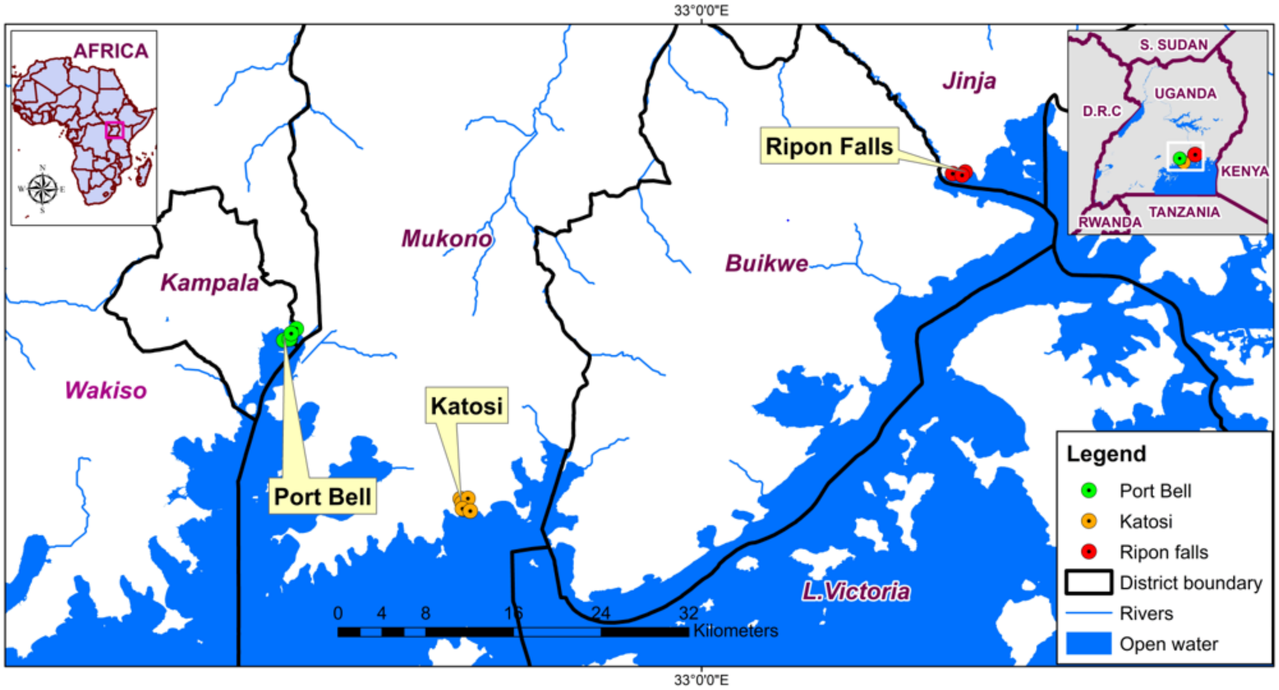

Fig. S1. Location of fish landing beaches on Lake Victoria where surface water was trawled. The points indicated per site are the transects towed during the sampling campaigns.

Table S1. Description of fish landing beaches where surface water was trawled on Lake Victoria

Site	Code	Description
Ripon Falls	RIFA	Named after the then undersecretary of state for India [George Frederick Samuel Robinson, 1st marquess of Ripon (1827–1909)]. It is located on the Victoria Nile, just below the river's outlet from L. Victoria, and was historically believed to be the source of River Nile. Part of it is a slum located within an urban setting of Jinja City with poor waste management practices. This site is situated in the Napoleon Gulf and close to the source of River Nile, with many recreational beaches and various tourist activities
Katosi	KAT	Situated in a rural area of Mukono district. Initially, Katosi was a cultural and tourist site but it now connects Koome Island in Mukono district to the Buvuma Islands of Tanzania. It is the most organized and the biggest fish landing center with most of the fish exported to Europe and the Middle East
Port Bell	PB	Port Bell is positioned at the end of a narrow inlet of L. Victoria (shores of Murchison Bay) in the vicinity of a busy trading center, an urban setting of Kampala city and part of the Luzira Industrial Park. It is both a fish landing beach and a freight port that links Kenya (Kisumu) and Tanzania (Mwanza) to Uganda

S2. Physiochemical properties of lake water varied among FLBs and between seasons

During the dry season, the lowest mean pH was 8.78±0.50 at Ripon Falls and the highest was 9.42±0.02 at Katosi. Surface water temperature ranged from 26.74±0.11°C at Ripon Falls to 27.42±0.40 °C at Katosi (Table S2). Electrical conductivity ranged from 75.50±0.71 µS/cm at Ripon Falls to 116.50±0.71 µS/cm at Port Bell. Salinity (0.03±0.00 mg/kg at Ripon Falls to 0.05±0.00 mg/kg at Port Bell), dissolved oxygen (7.15±0.55 mg/L at Katosi to 12.19±2.21 mg/L at Port Bell) and total dissolved solids (7.15±0.55 mg/L at Katosi to 12.19±2.21 mg/L at Port Bell) also varied considerably.

In the wet season, the pH (7.43±0.02 to 8.96±0.02), temperature (26.04±0.42°C to 27.67±0.13°C), electrical conductivity (74.00±0.00 µS/cm at Katosi to 102.50±2.12 µS/cm at Port Bell), salinity (0.03±0.00 mg/kg at Katosi to 0.05±0.01 mg/kg at Port Bell), dissolved oxygen (8.37±2.02 mg/L at Katosi to 14.49±1.42 mg/L at Port Bell) and total dissolved solids (47.00±0.00 mg/L at Katosi to 65.00±1.41 mg/L at Port Bell) were comparable to those recorded during the dry season. These

parameters, according to Kruskal–Wallis's test, varied significantly when the effect of seasons and sampling locations were considered (Table S2; Table S3).

The pH, temperature and dissolved oxygen of water measured in L. Victoria are comparable to 6.49–9.87, 25.50–28.92°C and 0.02–12.19 mg/L reported for water from the lake's Murchison Bay and Napoleon Gulf analyzed over a seven year period [6]. The physiochemical properties of L. Victoria water obtained are, however, lower than previously found in its Kenyan Nyanza Gulf [7, 8]. These are also lower than reported during the dry and wet seasons for samples from Tocagua and Luruaco lakes of Columbia [9, 10]. The current results are in agreement with a previous finding that the surface water of L. Victoria have warmed by almost 1.2°C in 82 years, with its dissolved oxygen greater than 7.3 mg/L [11]. The fluctuations observed could suggest that L. Victoria is under environmental stress [6]. In contrast to our results, a study in Luruaco Lake of Colombia found that there were no significant seasonal variations in the physicochemical characteristics of lake water during microplastic sampling campaigns [10].

Table S2. Non-conservable parameters of surface water recorded *in situ* along different transects on Lake Victoria

Season	Sampling site	Transect	pH	Temperature (℃)	Conductivity (µS/cm)	Salinity (mg/kg)	Dissolved oxygen (mg/L)	Total dissolved solids (mg/L)
Dry	Ripon Falls	1	9.43	26.66	78	0.04	8.61	43
			9.40	26.82	77	0.03	8.56	42
		2	9.19	27.27	75	0.03	7.82	41
			8.90	27.05	76	0.03	8.28	42
		3	8.93	27.08	76	0.03	8.29	42
			9.01	27.13	76	0.03	8.03	42
	Katosi	1	8.92	27.11	77	0.03	8.63	42
			8.96	27.13	77	0.03	8.96	42
		2	9.00	27.14	78	0.03	8.52	43
			9.12	27.7	78	0.04	7.53	43
		3	9.13	27.72	79	0.04	7.54	43
			8.42	26.51	72	0.03	6.76	40
	Port Bell	1	8.86	26.98	116	0.05	10.50	64
			9.13	27.06	117	0.05	10.89	64
		2	8.98	27.02	117	0.05	10.42	65
			9.04	27.02	116	0.05	10.61	64
		3	9.07	27.04	116	0.05	10.63	64
			9.43	27.37	111	0.05	13.75	61
Wet	Ripon Falls	4	8.94	27.76	80	0.03	11.11	50
			8.97	27.57	82	0.04	12.37	51
		5	8.99	27.58	82	0.04	12.31	51
			8.57	27.36	83	0.04	13.04	52
		6	8.47	27.31	82	0.04	11.75	51
			8.82	27.75	81	0.03	11.62	50

Season	Sampling site	Transect	pH	Temperature (℃)	Conductivity (µS/cm)	Salinity (mg/kg)	Dissolved oxygen (mg/L)	Total dissolved solids (mg/L)
	Katosi	4	7.70	26.44	75	0.03	6.94	47
			7.30	25.99	74	0.03	9.79	47
		5	7.37	25.58	74	0.03	13.27	48
			7.36	26.12	75	0.03	9.31	48
		6	7.44	26.33	74	0.03	7.59	47
			7.41	25.74	74	0.03	14.36	48
	Port Bell	4	7.42	25.88	75	0.03	13.44	48
			7.50	26.29	75	0.03	9.91	47
		5	7.51	26.34	75	0.03	9.58	47
			8.18	26.47	75	0.03	10.20	47
		6	8.37	26.08	104	0.05	13.48	66
			9.07	26.44	101	0.04	15.49	64
Kruskal-Walli's test			H = 22.89, P = 0.0004	H = 28.42, P<0.0001	H = 25.94, P<0.0001	H = 21.11, P = 0.0008	H = 20.43, P = 0.001	H = 29.44, P<0.001

Table S3. Dunn's post hoc test results for the physicochemical parameters of surface water from Lake Victoria

Parameter	Parameters	Mean Rank Diff	Z	Prob	Sig
pH	"Ripon Falls Dry Season" "Katosi Dry Season"	4.5	0.73989	1	0
	"Ripon Falls Dry Season" "Port Bell Dry Season"	0.5	0.08221	1	0
	"Ripon Falls Dry Season" "Ripon Falls Wet Season"	9.41667	1.54829	1	0
	**"Ripon Falls Dry Season" "Katosi Wet Season"**	**23.25**	**3.82277**	**0.00198**	**1**
	"Ripon Falls Dry Season" "Port Bell Wet Season"	15.83333	2.60332	0.13849	0
	"Katosi Dry Season" "Port Bell Dry Season"	-4	-0.65768	1	0
	"Katosi Dry Season" "Ripon Falls Wet Season"	4.91667	0.8084	1	0
	**"Katosi Dry Season" "Katosi Wet Season"**	**18.75**	**3.08288**	**0.03075**	**1**
	"Katosi Dry Season" "Port Bell Wet Season"	11.33333	1.86343	0.93603	0
	"Port Bell Dry Season" "Ripon Falls Wet Season"	8.91667	1.46608	1	0
	**"Port Bell Dry Season" "Katosi Wet Season"**	**22.75**	**3.74056**	**0.00275**	**1**
	"Port Bell Dry Season" "Port Bell Wet Season"	15.33333	2.52111	0.17548	0
	"Ripon Falls Wet Season" "Katosi Wet Season"	13.83333	2.27448	0.34406	0
	"Ripon Falls Wet Season" "Port Bell Wet Season"	6.41667	1.05503	1	0
	"Katosi Wet Season" "Port Bell Wet Season"	-7.41667	-1.21945	1	0
Temperature	"Ripon Falls Dry Season" "Katosi Dry Season"	-5.16667	-0.84956	1	0
	"Ripon Falls Dry Season" "Port Bell Dry Season"	0.25	0.04111	1	0
	"Ripon Falls Dry Season" "Ripon Falls Wet Season"	-11.41667	-1.87725	0.90726	0
	"Ripon Falls Dry Season" "Katosi Wet Season"	15.16667	2.49387	0.18954	0
	"Ripon Falls Dry Season" "Port Bell Wet Season"	12.66667	2.08279	0.55906	0

Parameter	Parameters	Mean Rank Diff	Z	Prob	Sig
	"Katosi Dry Season" "Port Bell Dry Season"	5.41667	0.89067	1	0
	"Katosi Dry Season" "Ripon Falls Wet Season"	-6.25	-1.02769	1	0
	**"Katosi Dry Season" "Katosi Wet Season"**	**20.33333**	**3.34342**	**0.01241**	**1**
	"Katosi Dry Season" "Port Bell Wet Season"	17.83333	2.93235	0.05046	0
	"Port Bell Dry Season" "Ripon Falls Wet Season"	-11.66667	-1.91836	0.82598	0
	"Port Bell Dry Season" "Katosi Wet Season"	14.91667	2.45276	0.21265	0
	"Port Bell Dry Season" "Port Bell Wet Season"	12.41667	2.04168	0.61775	0
	**"Ripon Falls Wet Season" "Katosi Wet Season"**	**26.58333**	**4.37112**	**1.85419E-4**	**1**
	**"Ripon Falls Wet Season" "Port Bell Wet Season"**	**24.08333**	**3.96004**	**0.00112**	**1**
	"Katosi Wet Season" "Port Bell Wet Season"	-2.5	-0.41108	1	0
Conductivity	"Ripon Falls Dry Season" "Katosi Dry Season"	-1.5	-0.24797	1	0
	**"Ripon Falls Dry Season" "Port Bell Dry Season"**	**-18.83333**	**-3.11346**	**0.02774**	**1**
	"Ripon Falls Dry Season" "Ripon Falls Wet Season"	-10.83333	-1.79093	1	0
	"Ripon Falls Dry Season" "Katosi Wet Season"	9.33333	1.54295	1	0
	"Ripon Falls Dry Season" "Port Bell Wet Season"	-1.16667	-0.19287	1	0
	"Katosi Dry Season" "Port Bell Dry Season"	-17.33333	-2.86548	0.06246	0
	"Katosi Dry Season" "Ripon Falls Wet Season"	-9.33333	-1.54295	1	0
	"Katosi Dry Season" "Katosi Wet Season"	10.83333	1.79093	1	0
	"Katosi Dry Season" "Port Bell Wet Season"	0.33333	0.05511	1	0
	"Port Bell Dry Season" "Ripon Falls Wet Season"	8	1.32253	1	0
	**"Port Bell Dry Season" "Katosi Wet Season"**	**28.16667**	**4.65641**	**<0.0001**	**1**

Parameter	Parameters	Mean Rank Diff	Z	Prob	Sig
	"Port Bell Dry Season" "Port Bell Wet Season"	17.66667	2.92059	0.05241	0
	**"Ripon Falls Wet Season" "Katosi Wet Season"**	**20.16667**	**3.33388**	**0.01285**	**1**
	"Ripon Falls Wet Season" "Port Bell Wet Season"	9.66667	1.59806	1	0
	"Katosi Wet Season" "Port Bell Wet Season"	-10.5	-1.73582	1	0
Salinity	"Ripon Falls Dry Season" "Katosi Dry Season"	-2.41667	-0.44671	1	0
	**"Ripon Falls Dry Season" "Port Bell Dry Season"**	**-19.58333**	**-3.61993**	**0.00442**	**1**
	"Ripon Falls Dry Season" "Ripon Falls Wet Season"	-7.25	-1.34014	1	0
	"Ripon Falls Dry Season" "Katosi Wet Season"	2.41667	0.44671	1	0
	"Ripon Falls Dry Season" "Port Bell Wet Season"	-3.66667	-0.67777	1	0
	**"Katosi Dry Season" "Port Bell Dry Season"**	**-17.16667**	**-3.17321**	**0.02261**	**1**
	"Katosi Dry Season" "Ripon Falls Wet Season"	-4.83333	-0.89343	1	0
	"Katosi Dry Season" "Katosi Wet Season"	4.83333	0.89343	1	0
	"Katosi Dry Season" "Port Bell Wet Season"	-1.25	-0.23106	1	0
	"Port Bell Dry Season" "Ripon Falls Wet Season"	12.33333	2.27978	0.33931	0
	**"Port Bell Dry Season" "Katosi Wet Season"**	**22**	**4.06664**	**7.15428E-4**	**1**
	**"Port Bell Dry Season" "Port Bell Wet Season"**	**15.91667**	**2.94215**	**0.04889**	**1**
	"Ripon Falls Wet Season" "Katosi Wet Season"	9.66667	1.78686	1	0
	"Ripon Falls Wet Season" "Port Bell Wet Season"	3.58333	0.66237	1	0
	"Katosi Wet Season" "Port Bell Wet Season"	-6.08333	-1.12449	1	0
	"Ripon Falls Dry Season" "Katosi Dry Season"	1.33333	0.2192	1	0
	"Ripon Falls Dry Season" "Port Bell Dry Season"	-15.16667	-2.49338	0.1898	0

Parameter	Parameters	Mean Rank Diff	Z	Prob	Sig
Dissolved Oxygen	**"Ripon Falls Dry Season" "Ripon Falls Wet Season"**	**-18.66667**	**-3.06878**	**0.03224**	**1**
	"Ripon Falls Dry Season" "Katosi Wet Season"	-8.66667	-1.42479	1	0
	"Ripon Falls Dry Season" "Port Bell Wet Season"	-16.83333	-2.76738	0.08476	0
	"Katosi Dry Season" "Port Bell Dry Season"	-16.5	-2.71258	0.10014	0
	**"Katosi Dry Season" "Ripon Falls Wet Season"**	**-20**	**-3.28798**	**0.01514**	**1**
	"Katosi Dry Season" "Katosi Wet Season"	-10	-1.64399	1	0
	**"Katosi Dry Season" "Port Bell Wet Season"**	**-18.16667**	**-2.98658**	**0.04232**	**1**
	"Port Bell Dry Season" "Ripon Falls Wet Season"	-3.5	-0.5754	1	0
	"Port Bell Dry Season" "Katosi Wet Season"	6.5	1.06859	1	0
	"Port Bell Dry Season" "Port Bell Wet Season"	-1.66667	-0.274	1	0
	"Ripon Falls Wet Season" "Katosi Wet Season"	10	1.64399	1	0
	"Ripon Falls Wet Season" "Port Bell Wet Season"	1.83333	0.3014	1	0
	"Katosi Wet Season" "Port Bell Wet Season"	-8.16667	-1.34259	1	0
Total Dissolved Solids	"Ripon Falls Dry Season" "Katosi Dry Season"	-1.5	-0.24844	1	0
	**"Ripon Falls Dry Season" "Port Bell Dry Season"**	**-26.25**	**-4.34777**	**2.06295E-4**	**1**
	**"Ripon Falls Dry Season" "Ripon Falls Wet Season"**	**-19.75**	**-3.27118**	**0.01607**	**1**
	"Ripon Falls Dry Season" "Katosi Wet Season"	-12.25	-2.02896	0.63694	0
	"Ripon Falls Dry Season" "Port Bell Wet Season"	-16.75	-2.77429	0.08298	0
	**"Katosi Dry Season" "Port Bell Dry Season"**	**-24.75**	**-4.09932**	**6.21538E-4**	**1**
	**"Katosi Dry Season" "Ripon Falls Wet Season"**	**-18.25**	**-3.02273**	**0.03758**	**1**
	"Katosi Dry Season" "Katosi Wet Season"	-10.75	-1.78051	1	0

Parameter	Parameters	Mean Rank Diff	Z	Prob	Sig
	"Katosi Dry Season" "Port Bell Wet Season"	-15.25	-2.52585	0.17313	0
	"Port Bell Dry Season" "Ripon Falls Wet Season"	6.5	1.07659	1	0
	"Port Bell Dry Season" "Katosi Wet Season"	14	2.31881	0.30608	0
	"Port Bell Dry Season" "Port Bell Wet Season"	9.5	1.57348	1	0
	"Ripon Falls Wet Season" "Katosi Wet Season"	7.5	1.24222	1	0
	"Ripon Falls Wet Season" "Port Bell Wet Season"	3	0.49689	1	0
	"Katosi Wet Season" "Port Bell Wet Season"	-4.5	-0.74533	1	0

Sig equals **1** indicates that the difference of the means is significant at the 0.05 level.

Sig equals 0 indicates that the difference of the means is NOT significant at the 0.05 level.

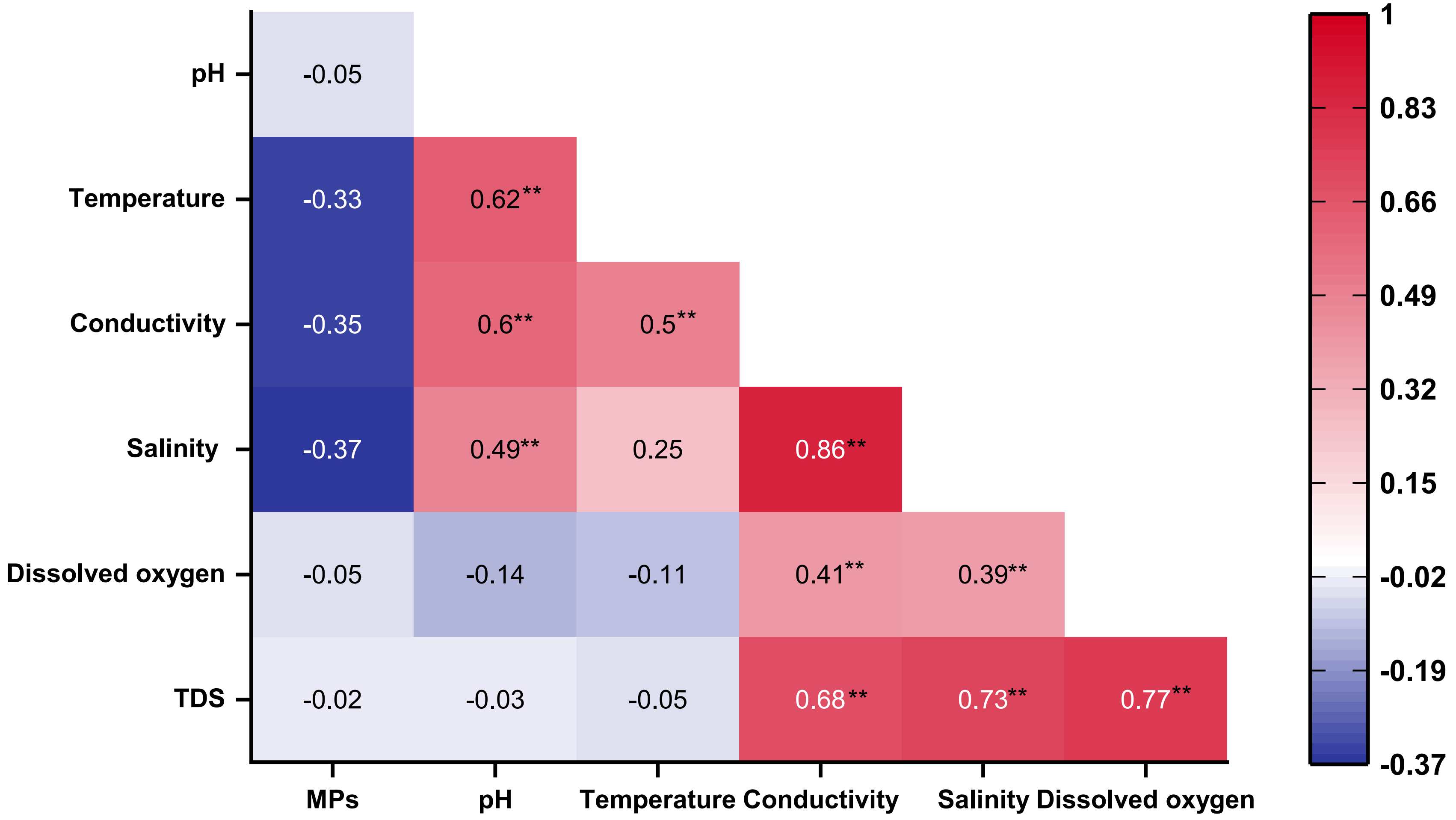

Fig. S2. Spearman's rank correlation coefficient matrix plot visualising the monotonic relationships between microplastics abundance (MPs) and the physicochemical properties of surface water. TDS = Total Dissolved Solids, ** Correlation is significant at the P<0.01 and P<0.05 levels (2-tailed). Colours indicate the strength and direction of Spearman's correlation coefficient (ρ).

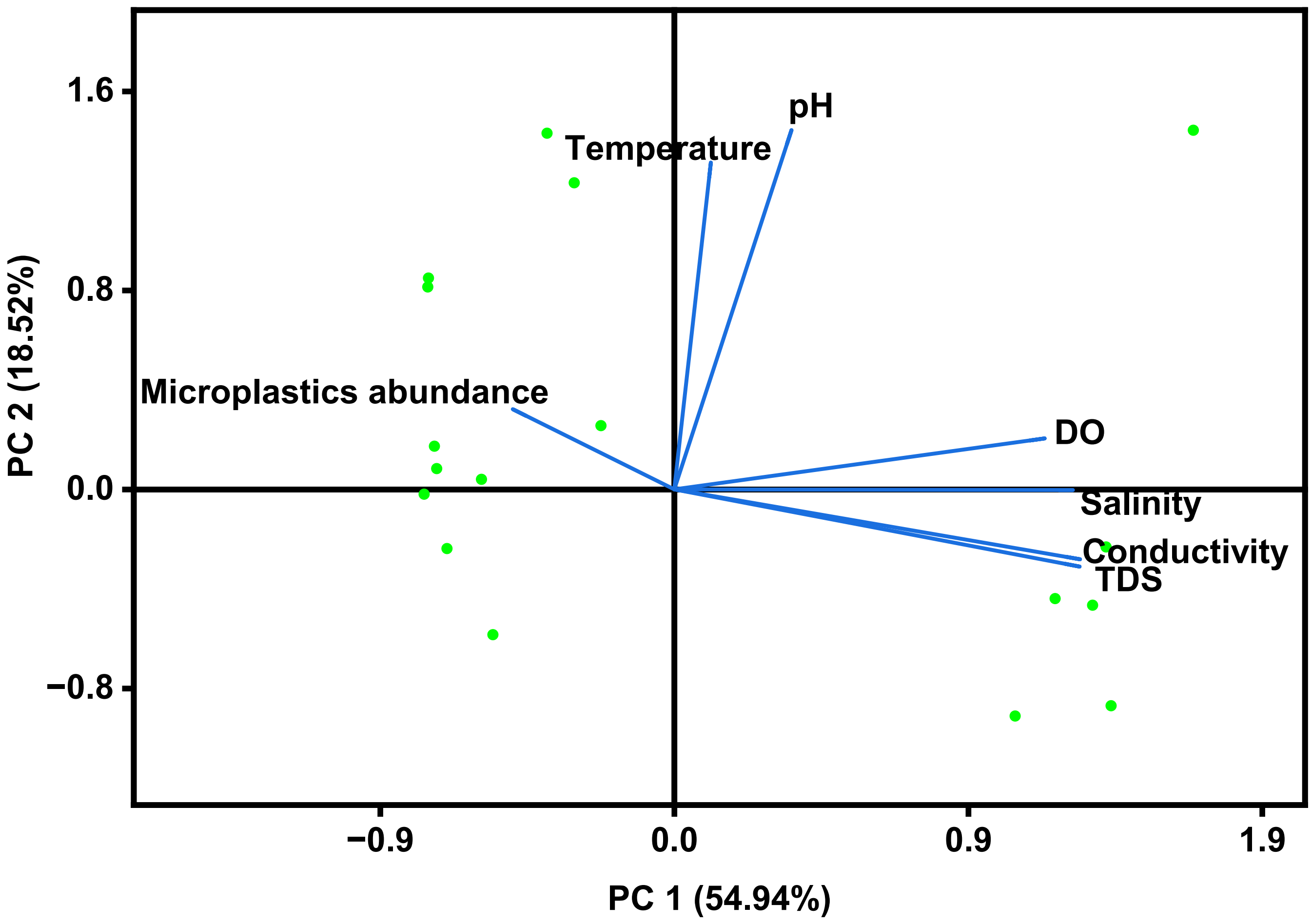

Fig. S3. Principal Component Analysis biplot loading of the standardized physicochemical parameters and microplastic abundance. Vectors indicate variable loadings, while points represent individual sampling transects across sites and seasons. DO = Dissolved oxygen, TDS = Total Dissolved Solids.

Table S4. Coordinates, area sampled, volume, and number of microplastics in surface water of L. Victoria

Season	Site	Transect	Sample ID	Coordinates		No. of revolutions	Tow length (km) [a]	Area sampled (km^2) [b]	Filtered water volume (m^3) [c]	No. of MPs
				Start	End					
Dry	Ripon Falls	1	RIFAW01	0°24′52″N 33°12′19″E	0°24′39″N 33°12′5″E	2618	0.5889	0.00017667	35.343	10
		2	RIFAW02	0°24′19″N 33°12′2″E	0°24′13″N 33°12′29″E	1877	0.8550	0.00025650	25.340	19
		3	RIFAW03	0°24′50″N 33°12′24″E	0°24′51″N 33°12′51″E	2373	0.8354	0.00025062	32.036	12
	Katosi	1	KATW01	0°9′1″N 32°48′8″E	0°9′2″N 32°48′30″E	1081	0.5398	0.00016194	14.594	15
		2	KATW02	0°8′49″N 32°48′13″E	0°8′32″N 32°48′14″E	2458	0.5001	0.00015003	33.183	16
		3	KATW03	0°8′31″N 32°48′16″E	0°8′25″N 32°48′37″E	2411	0.5398	0.00016194	32.549	10
	Port Bell	1	PBW01	0°16′49″N 32°39′26″E	0°16′53″N 32°39′43″E	874	0.6810	0.00020430	11.799	13
		2	PBW02	0°16′53″N 32°39′45″E	0°17′9″N 32°39′48″E	1087	0.5231	0.00015693	14.675	8
		3	PBW03	0°17′23″N 32°40′3″E	0°17′27″N 32°40′20″E	2074	0.6750	0.00020250	27.999	4
Wet	Ripon Falls	4	RIFAW04	0°24′16″N 33°11′58″E	0°24′5″N 33°11′30″E	4668	0.8680	0.00026040	63.0180	4
		5	RIFAW05	0°24′43″N 33°12′24″E	0°24′47″N 33°12′45″E	4767	0.6609	0.00019827	64.3545	10
		6	RIFAW06	0°24′3″N 33°12′33″E	0°24′5″N 33°12′55″E	4975	0.6830	0.00020490	67.1625	11
	Katosi	4	KATW04	0°9′1″N 32°48′8″E	0°9′2″N 32°48′30″E	1350	0.6810	0.00020430	18.2250	7
		5	KATW05	0°8′49″N 32°48′13″E	0°8′32″N 32°48′14″E	1879	0.5231	0.00015693	25.3665	11
		6	KATW06	0°8′31″N 32°48′16″E	0°8′25″N 32°48′37″E	1740	0.6750	0.00020250	23.4900	12
	Port Bell	4	PBW04	0°16′49″N 32°39′26″E	0°16′53″N 32°39′43″E	1650	0.5398	0.00016194	22.2750	11
		5	PBW05	0°16′53″N 32°39′45″E	0°17′9″N 32°39′48″E	1654	0.5001	0.00015003	22.3290	7
		6	PBW06	0°17′23″N 32°40′3″E	0°17′27″N 32°40′20″E	1661	0.5398	0.00016194	22.4235	11

[a] Tow length (km) was calculated using the coordinates.

[b] Area = Tow length (km) × width of manta trawl (30 cm = 0.0003 km)

[c] Volume of water filtered by manta net = number of revolutions × 0.3 × net opening area (0.3 × 0.15 m^2) × 1000 (litres).

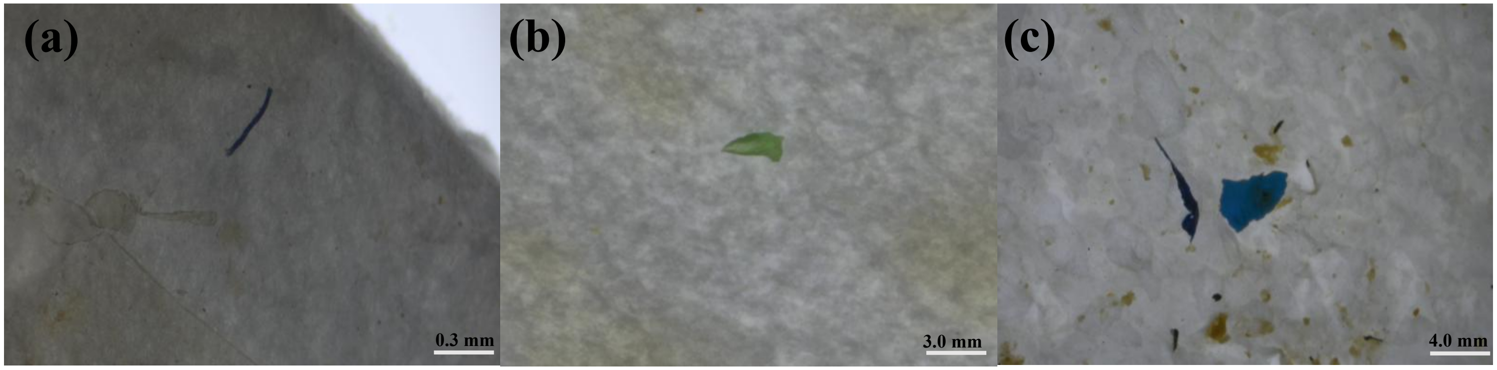

Fig. S4. Micrographs of plastic particles isolated from surface water of Lake Victoria (a) fiber, (b) and (c) fragments.

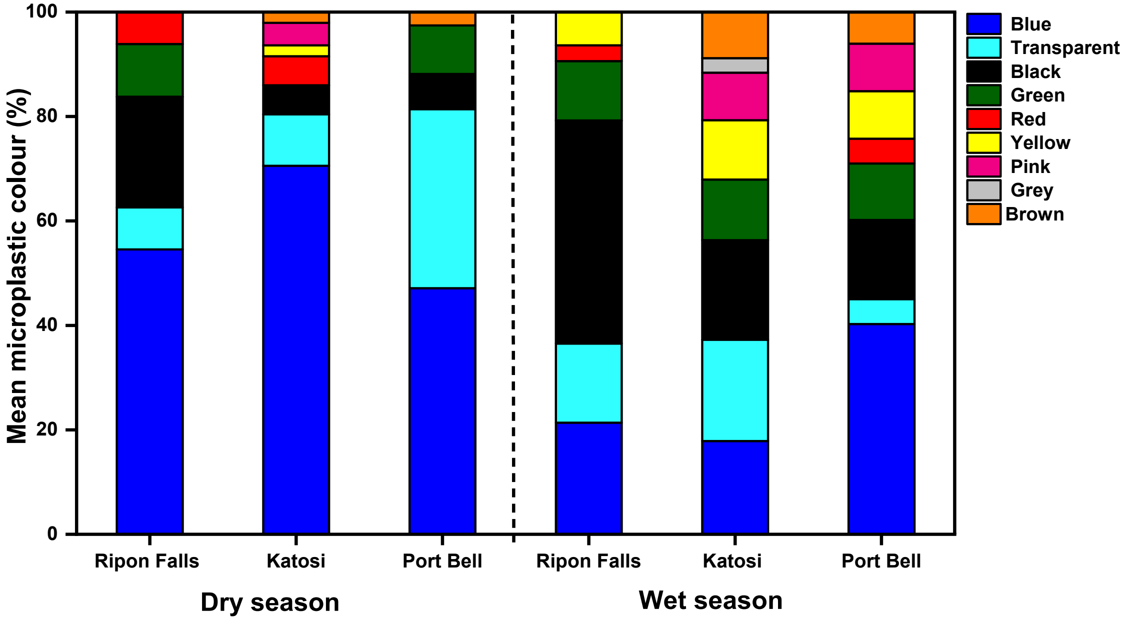

Fig. S5. Microplastic colours in surface water of Lake Victoria

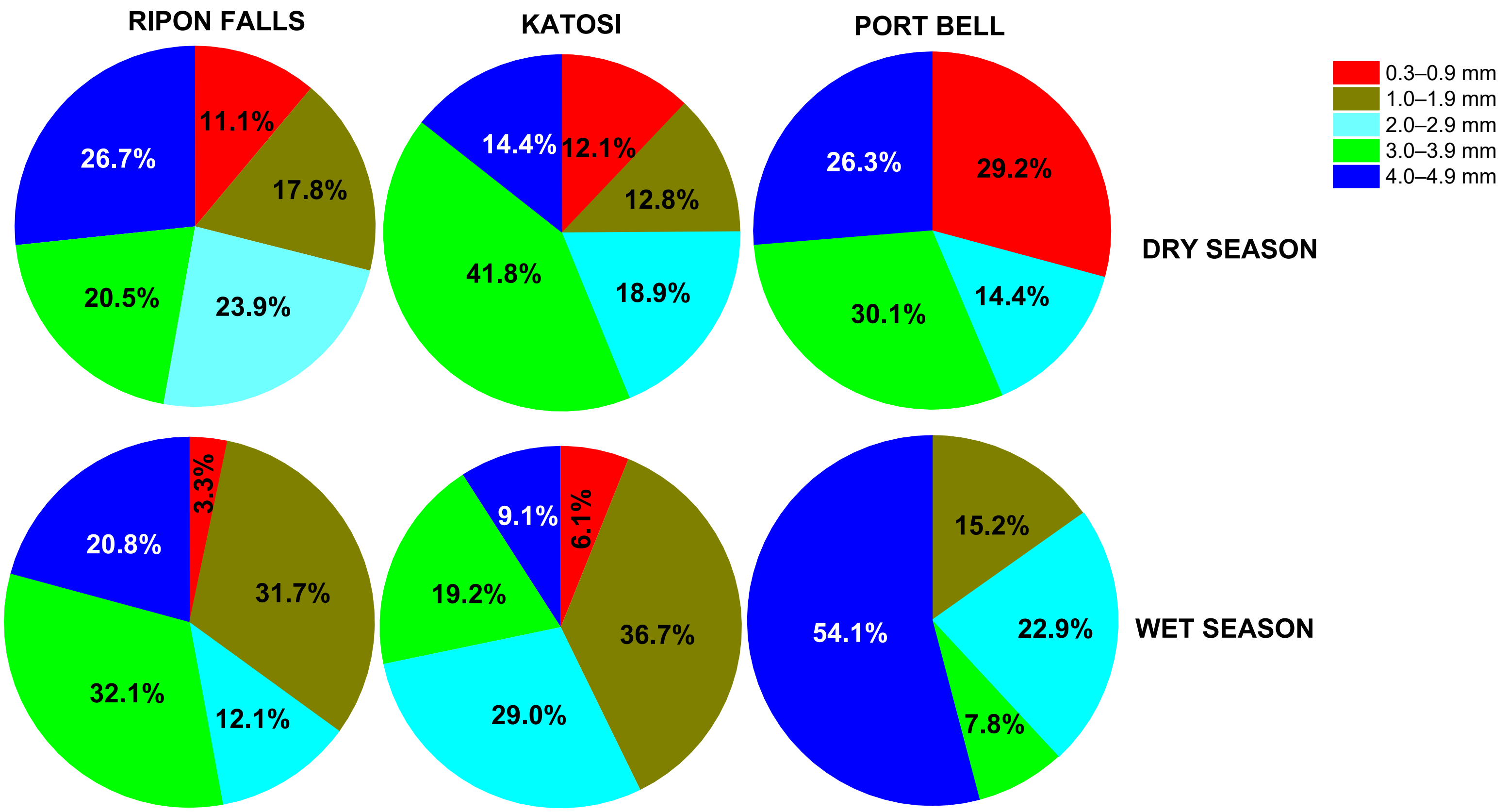

Fig. S6. Size distribution of microplastics in surface water from Lake Victoria

Table S5. Polymers identified by μ-FTIR in surface water samples from L. Victoria

Fish landing beach	Field code	μ-FTIR No.	Spectrum No.	Polymers identified	Scores (%)
Ripon Falls (dry season)	RIFAW03	01	01 FTIR-1	Polyamide (Nylon 6)	63.7
			01 FTIR-2	Polyamide (Nylon 6)	63.6
			01 FTIR-3	Polyacrylamide	66.1
			01 FTIR-4	Polypropylene	69.9
			01 FTIR-5	Polyethylene	74.5
			01 FTIR-6	Polypropylene	68.3
			01 FTIR-7	Polypropylene	79.7
Ripon Falls (wet season)	RIFAW04	02	02 FTIR-1	Polyamide (Nylon 6/66)	57.6 [a]
			02 FTIR-2	Polyethylene	93.3
			02 FTIR-3	Polyethylene/polypropylene copolymer	70.4
			02 FTIR-4	Polyethylene/polypropylene copolymer	70.5
Katosi (dry season)	KATW03	03	03 FTIR-1	Polypropylene	91.9
			03 FTIR-3	Polyethylene	95.5
			03 FTIR-4	Polyacetylene	69.2[b]
			03 FTIR-5	Polyacetylene	67.4[b]
Katosi (wet season)	KATW05	04	04 FTIR-1	Polyethylene	96.9
			04 FTIR-2	Polyethylene	94.3
			04 FTIR-3	Polyacrylic acid	56.5 [a]
			04 FTIR-4	Polyacrylic acid	56.4 [a]
Port Bell (dry season)	PBW03	05	05 FTIR-1	Polyamide (Nylon 6)	62.8

Fish landing beach	Field code	µ-FTIR No.	Spectrum No.	Polymers identified	Scores (%)
			05 FTIR-2	Polyethylene	90.8
			05 FTIR-3	Polyethylene	91.0
			05 FTIR-4	Polypropylene	68.5
Port Bell (wet season)	PBW06	06	06 FTIR-1	Polyethylene	73.2
			06 FTIR-2	Polyacrylamide	66.9
			06 FTIR-3	Polyacrylamide	67.0
			06 FTIR-4	High Density Polyethylene	86.9
			06 FTIR-5	Polyacrylamide	67.1
			06 FTIR-6	Polypropylene	79.7

Note: [a] Excluded since they had scores less than 60%.

[b] Peaks are not consistent with peaks of any known stable synthetic polymers. Polyacetylene is unstable in the environment. Samples for Pyr-GC-MS were RIFAW01, RIFAW02, KATW01, KATW02, PBW01, PBW02 and RIFAW05, RIFAW06, KATW04, KATW06, PBW04 and PBW05. These corresponds to Ripon Falls 01, Ripon Falls 02, Katosi 01, Katosi 02, Port Bell 01, Port Bell 02, Ripon Falls 03, Ripon Falls 04, Katosi 03, Katosi 04, Port Bell 03 and Port Bell 04 listed in **Table 3**.

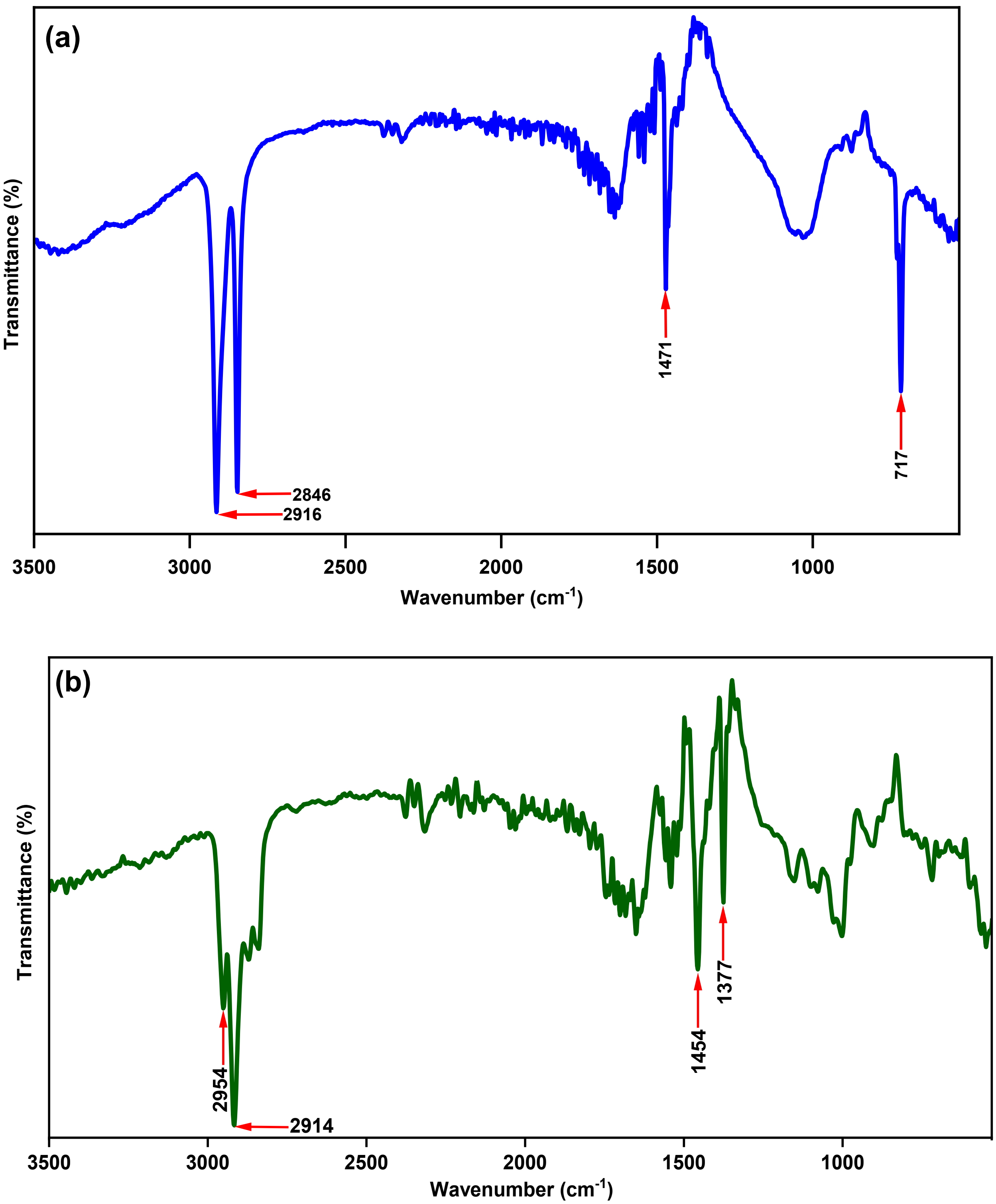

Fig. S7. μ-FTIR spectra of (a) polyethylene, and (b) polypropylene particles acquired in attenuated total reflectance mode. Wavenumbers of prominent bands are indicated by arrows.

Table S6. Potential interferences and other materials pyrolysed to assess the selectivity of diagnostic indicator pyrolysates (values are in µg)

Potential interferent/matrix/control	Composition	PE	PP	PS	ABS	SBR	PMMA	PC	PET	PVC	N6	N66
Blank (new empty Eco-cup, *n* = 5)	Deactivated stainless steel	<LOD	<LOD	<LOD	<LOD	<LOD	<LOD	<LOD	<LOD	<LOD	<LOD	<LOD
Quartz wool (in a blank cup)	Quartz wool	<LOD	<LOD	<LOD	<LOD	<LOD	<LOD	<LOD	<LOD	<LOD	0.6	<LOD
Microfiber glass filter material (2 discs)	Borosilicate glass fibers	<LOD	<LOD	<LOD	<LOD	<LOD	<LOD	0.4	<LOD	<LOD	<LOQ	<LOD
Nitrile gloves (Lab Logistics Group, Meckenheim, Germany; 0.25 mg)	Nitrile butadiene rubber	<LOD	<LOD	<LOD	<LOD	<LOD	<LOD	<LOD	<LOD	<LOD	<LOQ	<LOD
Water bottle (0.17 mg) [a]	PET and additives	<LOD	225.2	133.1	<LOD	14.3	<LOD	<LOD	1.8	<LOD	<LOQ	<LOD
Swab tip of cotton buds (Agilent item: D7607-100; 0.25 mg)	Cotton fibers/natural cellulose	<LOD	<LOD	<LOD	<LOD	<LOD	<LOD	<LOD	<LOD	<LOD	<LOQ	<LOD
Soda lignin (0.15 mg)	Lignin	228.9	<LOD	2.9	<LOD	623.2	<LOD	<LOD	20.5	10.9	4.2	4.8
Indulin AT (0.33 mg)	Wood/straw	<LOD	<LOD	<LOD	<LOD	<LOD	<LOD	<LOD	<LOD	<LOD	0.5	<LOD
Polystyrene reference material (50 µg/µL)	Polystyrene thin film in methyl stearate	<LOD	277.6	163.8	<LOQ	<LOD	<LOD	<LOD	10.7	<LOD	<LOQ	<LOD
ABS filament (1.61 mg) [b]	Acrylonitrile butadiene styrene	37.0	1950.1	1144.4	14.6	<LOD	2.1	<LOD	69.7	1383.7	15.1	17.7
PLA filament (Netherlands; 1.70 mg) [b]	Polylactic acid	29.4	<LOD	2.9	<LOD	209.4	1.3	<LOD	6.7	34.9	291.5	345.4
PET-G filament (Netherlands; 0.35 mg) [b]	PET glycol	235.1	9.8	6.7	4.8	1186.0	2.4	<LOD	10.7	5.9	1.2	1.3
Polypropylene filament (0.68 mg) [b]	Polypropylene	562.3	20.4	12.9	<LOD	83.8	41.8	<LOD	2.3	5.7	858.7	1017.7
HIPS filament chips (China; 1.25 mg) [b]	Polystyrene and rubber [c]	107.0	2229.6	5223.7	97.8	5449.8	<LOD	<LOD	27.5	153.5	7.2	8.3
Cotton swab tip (CMC Consumer Care, Germany; 0.25 mg)	Cotton fibers/cellulose	<LOD	<LOD	<LOD	<LOD	<LOD	<LOD	<LOD	<LOD	<LOD	<LOQ	<LOD
Blue barrel (unknown origin; 0.43 mg)	High density PE	8248.2	23.8	14.8	<LOD	<LOD	24.7	<LOD	1.7	8.3	293.7	347.9

[a] Carbonated natural mineral water-green bottle, Austria (Römerquelle pricklend brand).

[b] 3D printing filaments.

[c] The rubber or polybutadiene additives are grafted into the polystyrene matrix.

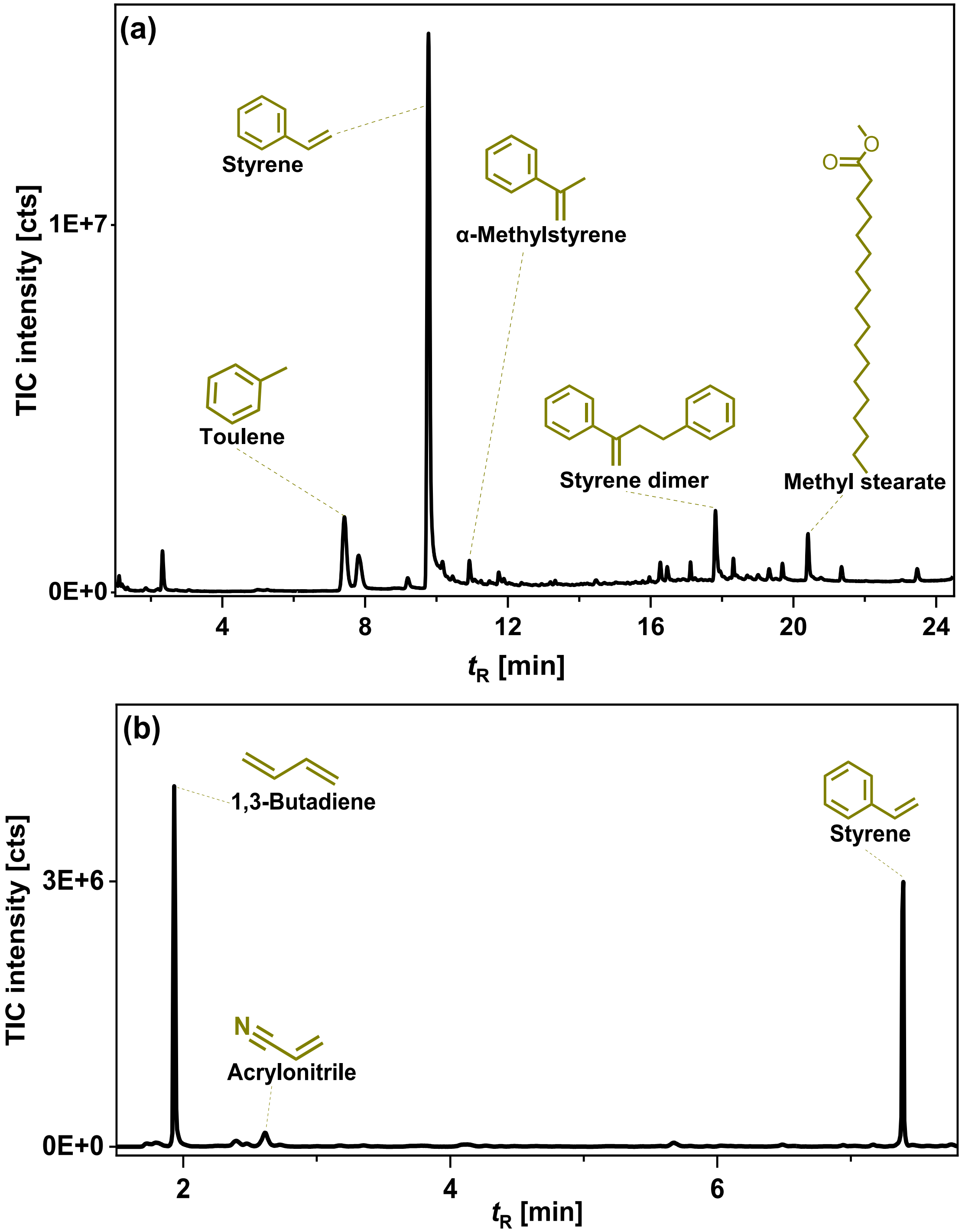

Fig. S8. Pyrograms of other analytical quality assurance materials pyrolysed (a) polystyrene reference material (as a continuing calibration-check sample, t_R = 10.081 min was relocked to styrene), and (b) nitrile glove.

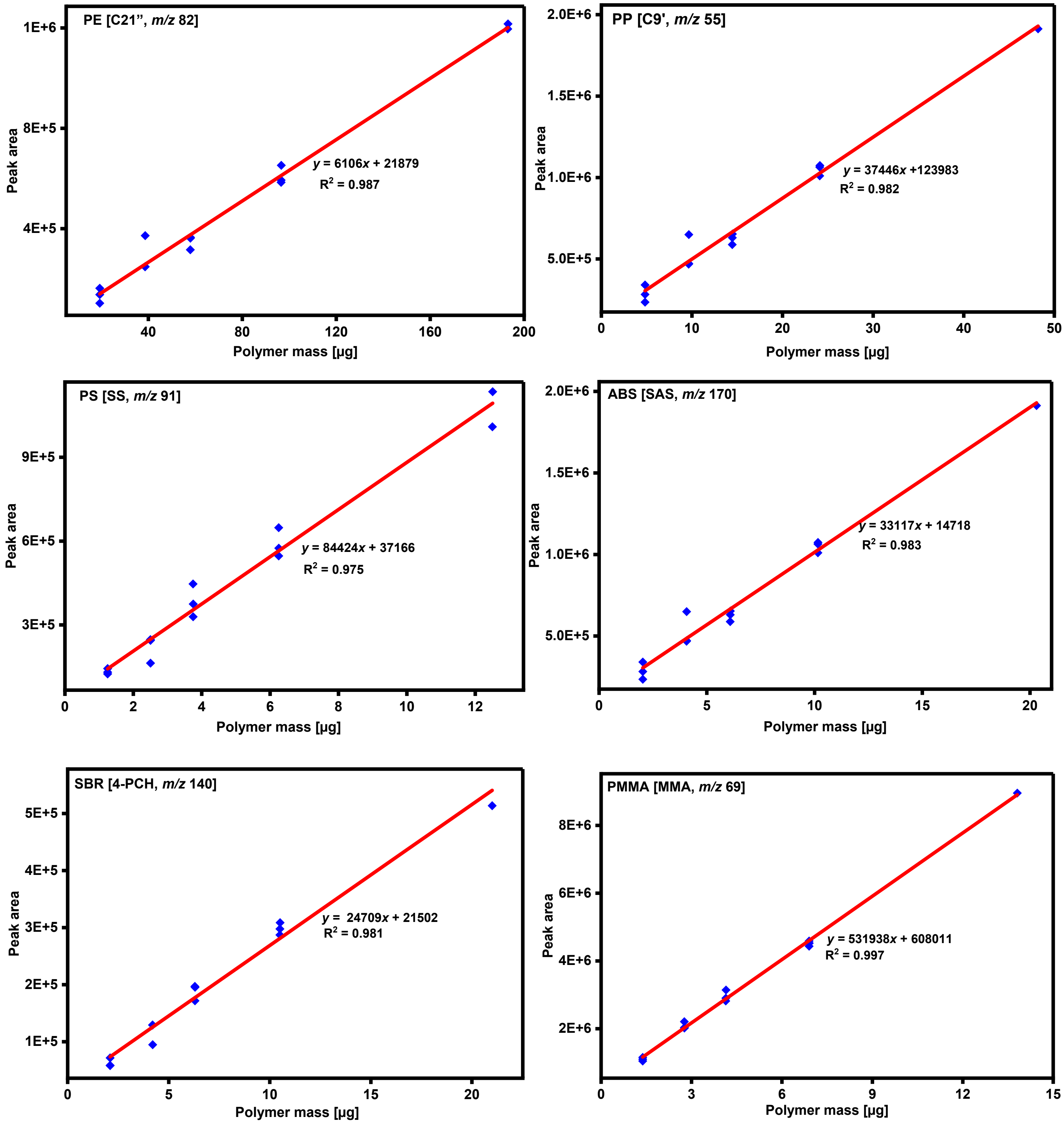
PE [C21”, m/z 82]
y = 6106x + 21879
R2 = 0.987
Peak area
1E+6
8E+5
4E+5
40
80
120
160
200
Polymer mass [µg]
PP [C9', m/z 55]
y = 37446x +123983
R2 = 0.982
Peak area
2.0E+6
1.5E+6
1.0E+6
5.0E+5
0
10
20
30
40
50
Polymer mass [µg]
PS [SS, m/z 91]
y = 84424x + 37166
R2 = 0.975
Peak area
9E+5
6E+5
3E+5
0
2
4
6
8
10
12
Polymer mass [µg]
ABS [SAS, m/z 170]
y = 33117x + 14718
R2 = 0.983
Peak area
2.0E+6
1.5E+6
1.0E+6
5.0E+5
0
5
10
15
20
Polymer mass [µg]
SBR [4-PCH, m/z 140]
y = 24709x + 21502
R2 = 0.981
Peak area
5E+5
4E+5
3E+5
2E+5
1E+5
0
5
10
15
20
Polymer mass [µg]
PMMA [MMA, m/z 69]
y = 531938x + 608011
R2 = 0.997
Peak area
8E+6
6E+6
4E+6
2E+6
0
3
6
9
12
15
Polymer mass [µg]

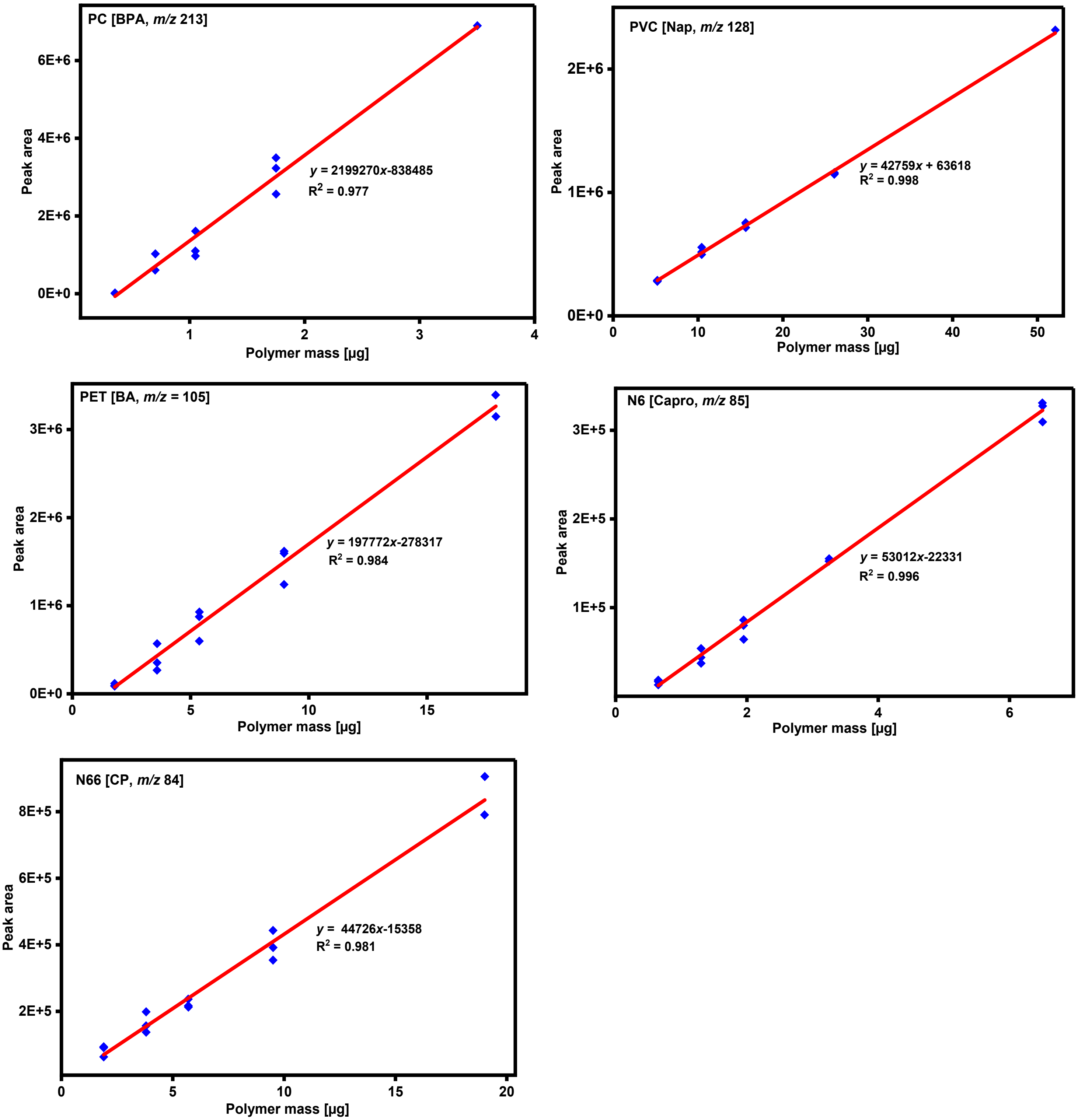

Fig. S9. Fitted calibration curves of the microplastic polymers. Outliers identified in MATLAB were excluded. The labels indicate polymers, their index compounds and quantification ions (in square brackets, as defined in Table 1).

References

1. Evans H. Setting sail on Lake Victoria to beat plastic pollution. Retrieved on March 20th, 2022 from https://www.theflipflopi.com/blog/setting-sail-on-lake-victoria-to-beat-plastic-pollution
2. Aura CM, Humphrey OS, Marriott AL, Watts MJ, Ongore CO, Mwamburi JM, Osano O, Coffey TJ. Assessing the spatial distribution of elemental concentrations in surface sediments of Lake Victoria, Kenya: implications for ecological health and management. *Environ Geochem Health* 2024; 46:137.
3. Nyamweya C, Lawrence TJ, Ajode MZ, Smith S, Achieng AO, Barasa JE, Masese FO, Taabu-Munyaho A, Mahongo S, Kayanda R, Rukunya E, Kisaka L, Manyala J, Medard M, Otoung S, Mrosso H, Sekadende B, Walakira J, Mbabazi S, Kishe M, Shoko A, Dadi T, Gemmell A, Nkalubo W. Lake Victoria: Overview of research needs and the way forward. *J Great Lakes Res*. 2023; 49: 102211.
4. World Lake Database. Lake Victoria. Retrieved on 21st August 2023 from https://wldb.ilec.or.jp/Display/html/3586
5. Egessa R, Nankabirwa A, Ocaya H, Pabire WG. Microplastic pollution in surface water of Lake Victoria. *Sci Total Environ.* 2020; 741: 140201.
6. Nalumenya B, Rubinato M, Catterson J, Kennedy M, Bakamwesiga H, Wabwire D. Assessing the Potential Impacts of Contaminants on the Water Quality of Lake Victoria: Two Case Studies in Uganda. *Sustainability* 2024; 16: 9128.
7. Orina P, Onyango D, Lungayia H, Oduor A, Sifuna A, Otuya P, Owigar R, Kowenje C, Hinzano S. Water Quality of Selected Fishing Beaches of Lake Victoria Kenyan Gulf. *Open J Ecol.* 2020; 10: 22-35.
8. Simiyu B, Amukhuma H, Sitoki L, Okello W, Kurmayer R. Interannual variability of water quality conditions in the Nyanza Gulf of Lake Victoria, Kenya. *J. Great Lakes Res*. 2022; 48: 97-109.
9. Miranda-Peña L, Urquijo M, Arana VA, García-Alzate R, García-Alzate CA, Trilleras J. Microplastics Occurrence in Fish from Tocagua Lake, Low Basin Magdalena River, Colombia. *Diversity* 2023; 15: 821.
10. Rojas-Luna RA, Oquendo-Ruiz L, García-Alzate CA, Arana VA, García-Alzate R, Trilleras J. Identification, Abundance, and Distribution of Microplastics in Surface Water Collected from Luruaco Lake, Low Basin Magdalena River, Colombia. *Water* 2023; 15: 344.
11. Sitoki L, Gichuki J, Ezekiel C, Wanda F, Mkumbo OC, Marshall BE. The Environment of Lake Victoria (East Africa): Current Status and Historical Changes. *Int Rev Hydrobiol*. 2010; 95:209-223.
